# Patient Education and Self‐Management in Adults With Temporomandibular Disorders: Results From a Systematic Review With Meta‐Analysis

**DOI:** 10.1111/joor.70187

**Published:** 2026-03-19

**Authors:** Geneviève Ferland, Raphaël Vincent, François Desmeules, Audrey‐Anne Cormier, Moira Huon, Laurent Pitance

**Affiliations:** ^1^ École De Réadaptation, Université De Montréal Montréal Canada; ^2^ Centre De Recherche de L'hôpital Maisonneuve‐Rosemont Montréal Canada; ^3^ Université Catholique De Louvain Louvain Belgium; ^4^ Institute of Experimental and Clinical Research, Health Sciences Division, Neuro‐Musculo‐Skeletal‐Lab (NMSK), UCLouvain Brussels Belgium; ^5^ Oral and Maxillofacial Department, Cliniques Universitaires Saint‐Luc Brussels Belgium

**Keywords:** education and self‐management, rehabilitation, temporomandibular disorders

## Abstract

**Objective:**

To evaluate the efficacy of education and self‐management (ED and SM) interventions delivered by a health care provider, either alone or in combination with other non‐surgical interventions for improving pain, function, or health‐related quality of life (HRQoL) in adults with temporomandibular disorders (TMDs).

**Design:**

Systematic review with meta‐analysis.

**Literature Search:**

Six databases were searched up to March 2025.

**Study Selection Criteria:**

Randomized clinical trials (RCTs) comparing the efficacy of ED and SM with any non‐surgical other interventions such as splints, manual therapy, electrotherapy or multimodal approaches in adults with TMDs. Eligible studies had to report outcomes on pain, function or HRQoL.

**Data Synthesis:**

Risk of bias was assessed using the Cochrane RoB‐1 tool. Pooled treatment effects were calculated using random‐effects models with standardized mean differences. Certainty of evidence was evaluated using the Grading of Recommendations Assessment, Development and Evaluation (GRADE) framework.

**Results:**

Forty‐seven RCTs (*n* = 3238 participants; 77% female; mean age: 34.4 ± 7.3 years) were included, and none had a low risk of bias. ED and SM interventions were generally delivered as standardized programs and often served as control conditions. Based on very low‐certainty evidence, other non‐surgical interventions may be more effective than ED and SM alone for short‐term pain reduction (SMD = 0.67, 95% CI: 0.13–1.20, 6 studies, 323 patients) or HRQoL improvement (SMD = 0.61, 95% CI: 0.20–1.01, 2 studies, 124 patients). When combined with ED and SM, non‐surgical interventions may also result in moderate additional HRQoL improvements (SMD = 0.50, 95% CI: 0.18–0.82, 3 studies, 154 patients). Other comparisons—such as ED and SM alone versus ED and SM combined with other non‐surgical interventions—showed comparable effects between groups on pain, function and HRQoL over the short‐, medium‐ and long‐term. However, the certainty of evidence supporting these findings remains low to very low.

**Conclusion:**

The certainty of evidence supporting ED and SM interventions for TMDs is very low to low across all outcomes. While some short‐term clinically relevant benefits may favour other non‐surgical treatments, no consistent superiority or inferiority of ED and SM was found. Combining ED and SM with other interventions did not consistently improve outcomes. High‐quality trials are needed to determine the effectiveness of ED and SM and their optimal delivery in TMD care.

**Prospero:**

CRD42024529862.

## Graphical abstract

Graphical abstract summarizing the evidence from randomized controlled trials comparing education and self‐management with other non‐surgical interventions for temporomandibular disorders.

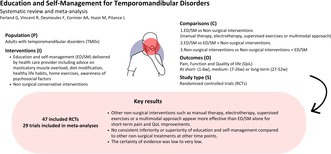

## 1 Introduction

Temporomandibular disorders (TMDs) are the leading cause of non‐dental orofacial pain and are considered the second most prevalent musculoskeletal disorder after chronic low back pain [1, 2, 3]. Individuals with TMD can experience a wide range of symptoms, such as pain in the temporomandibular joint (TMJ) region, the masticatory muscles, the inner or outer ear, and the neck. Adults may complain of joint noises and difficulty fully opening the mouth, leading to disability with mouth opening, chewing food, or talking [1, 2, 3]. Collectively, pain, associated symptoms and disabilities significantly impact the health‐related quality of life (HRQoL) of individuals suffering from TMDs [4, 5, 6].

TMDs are recognized as complex and multifactorial conditions, influenced by risk factors such as somatization, mood disorders, parafunctional habits, stress, and pain sensitivity [7, 8, 9, 10, 11, 12, 13, 14]. They are also frequently associated with comorbidities such as depression, anxiety, cervical spine dysfunction and headaches. Considering this complexity, TMDs are best understood and managed within a biopsychosocial framework [15].

Management of TMDs often involves multiple healthcare providers, including dentists, physiotherapists and physicians [16]. While dentists often prioritize the use of occlusal splints, empirical data regarding their effectiveness in reducing pain and disability remains inconclusive [17, 18]. In contrast, current clinical guidelines strongly advocate for multimodal interventions, which may include patient education, reassurance, relaxation therapy, manual therapy and exercises [1, 19, 20, 21].

Several systematic reviews have examined the effects of patient education (ED) and self‐management (SM) strategies in individuals with TMDs, and based on low to moderate quality trials, authors concluded that ED and SM may effectively reduce pain and disability and improve HRQoL [22, 23]. Furthermore, such interventions may yield outcomes comparable to other conservative treatments, such as supervised physiotherapy and manual therapies, occlusal splints or other combined multimodal therapeutic approaches. However, these earlier reviews lacked a consistent definition of ED and SM, as described in more recent conceptual frameworks. In particular, a Delphi consensus study [24] and a recent scoping review [25] have helped clarify the definition, structure and goals of ED and SM interventions for TMDs, identifying key therapeutic elements such as education on general TMD mechanisms, masticatory muscle overuse, postural awareness, psychosocial contributors and therapeutic exercises.

Furthermore, to date, no meta‐analysis has systematically appraised the effectiveness of ED and SM strategies compared with other non‐surgical treatments for TMDs. Therefore, the objective of this systematic review and meta‐analysis is to evaluate the effectiveness of education and self‐management interventions, including unsupervised home exercise programs, on pain, disability and HRQoL in adults with temporomandibular disorders, while also mapping the interventions against established definitions and thematic components.

## 2 Methods

This systematic review was conducted according to the Preferred Reporting Items for Systematic Reviews and Meta‐Analyses (PRISMA) guidelines. The study protocol was registered with PROSPERO in March 2024 and is available online (record number: CRD42024529862). https://www.crd.york.ac.uk/PROSPERO/view/CRD42024529862, https://www.crd.york.ac.uk/PROSPERO/display_record.php?RecordID.

### 2.1 Search Strategy

Multiple electronic searches were performed in bibliographic databases (MEDLINE, PubMed, Embase, Cochrane Central, CINAHL and PsycINFO) up to March 2025 with no restrictions on publication date [26]. See File S1 for details on the search strategy used.

The PICOS framework was employed to identify keywords and search terms based on the review question: *What is the efficacy of patient education including advice and unsupervised exercise programs compared with other conservative treatments in adults with temporomandibular disorders?* An information specialist assisted with the development of search terms on search implementation. Descriptors such as *temporomandibular joint disorders*, *temporomandibular joint diseases, counselling, self‐management, self‐care, exercises* and *patient education* as topics were used, as well as free‐text terms related to temporomandibular joint disorders, ED and SM. The PICOS criteria are the following:

- Population: Adults with temporomandibular disorders according to the Research Diagnostic Criteria for Temporomandibular Disorders (RDC/TMD), or its most recent version, the Diagnostic Criteria for Temporomandibular Disorders (DC/TMD) [27].
- Interventions: Interventions had to align with either one of the themes of ED and SM defined by Durham et al. [24] and van der Meer et al. [25].
- Comparators: Any non‐surgical treatment such as occlusal splint, medication, manual therapy (i.e., joint mobilization, joint manipulation, massage or myofascial techniques), electrotherapy (i.e., high voltage stimulation, therapeutic ultrasound, laser therapy), supervised exercise sessions, or any multimodal combination of the above‐mentioned interventions.
- Outcomes: Any patient‐related outcomes measuring pain, disability, or HRQoL.
- Study design: Randomized controlled trials.

### 2.2 Study Selection

Before commencing the dual‐reviewer screening process, the Cochrane RCT Classifier in Covidence was used to preliminarily exclude records that were clearly not randomized controlled trials. This highly sensitive tool greatly reduced the volume of irrelevant studies and streamlined the subsequent manual screening [28].

Two reviewers (MH and RV or GF and RV) independently screened all titles and abstracts. During the initial screening stage, the two reviewers reached consensus on study inclusion or exclusion when eligibility was unclear. Subsequently, they independently performed a full‐text screening of the selected studies. Any disagreements regarding eligibility were resolved through discussion; if consensus could not be reached, a third reviewer (LP or FD) was consulted to make the final decision.

Studies were eligible for inclusion if they met the following criteria: 1. Participants were adults with a diagnosed TMD according to the RDC/TMD or DC/TMD [27]; 2. The interventions consisted of education and advice for self‐management, as defined by Durham et al. [24] and van der Meer et al. [25] and delivered either as standalone approaches or in combination with other treatments. These included information about temporomandibular disorders, guidance on the jaw muscles and joints, postural education, awareness of lifestyle and psychosocial factors, unsupervised therapeutic exercises and other relevant educational content. Interventions could be delivered by a physiotherapist, dentist, or chiropractor and were compared to any other conservative treatment; 3. at least one outcome measured was related to pain, disability, or health‐related QoL; 4. the study design was an RCT published either in English or in French. Studies involving participants suffering from dysphagia conditions, tinnitus or migraine were excluded. Trials were excluded if they evaluated the effect of cognitive behavioural treatments delivered by psychologists or if advanced training in such therapeutic approaches was required by the health care provider involved. This decision was made to ensure that the review focused on simple, accessible and broadly implementable interventions that can be delivered by a wide range of health professionals in routine clinical settings.

### 2.3 Data Extraction

Data from the included studies were extracted using a standardized form that captured the following information: author names, year of publication, country of origin, care setting, classification of temporomandibular disorder diagnosis, participant characteristics and sample size, type of healthcare providers delivering the intervention, detailed description of the interventions and comparators, outcome measures, follow‐up duration and results. ED and SM interventions delivered in each trial were also coded against components from Durham et al. [24] and van der Meer et al. [25] by extracting all reported interventions in each included trial. Data extraction was conducted and independently verified by two evaluators (R.V. and G.F.). Disagreements were resolved through discussion.

The included trials were categorized for potential pooling and analysis, and further subgrouped by outcome measures (pain, disability and QoL) and by follow‐up time periods (short‐, medium‐, and long‐term):

- ED and SM alone compared to another intervention.
- Another intervention combined with ED and SM compared to the same intervention alone.
- ED and SM combined with another intervention compared to ED and SM alone.

### 2.4 Methodological Quality and Risk of Bias

The methodological quality of included studies was assessed with the Cochrane Risk of Bias tool version 1 (RoB 1) [29]. This tool assesses seven sources of potential bias: random sequence generation, allocation concealment, selective reporting, other sources of bias, personnel and participants blinding, outcome assessment blinding and incomplete outcome data. The methodological quality and risk of bias appraisal were assessed independently by two pairs of evaluators (M.H/R.V or G.F/AA.C) with final scores determined through consensus. In case of disagreement, an additional reviewer was consulted to help achieve consensus (L.P or R.V).

### 2.5 Data Analysis

Random‐effects model meta‐analyses were performed using Review Manager (RevMan 5.4, The Cochrane Collaboration, Copenhagen, Denmark). Since different tools were used to assess similar outcomes, the pooled effects of ED and SM were expressed as standardized mean differences (SMDs). For all meta‐analyses, alpha levels were set at 0.05% and 95% confidence intervals (CIs) were calculated. Studies were analysed based on the comparators and categorized into short (1–6 weeks), medium (7–26 weeks) and long‐term (27–52 weeks). A negative SMD indicates a beneficial effect on pain, disability, or HRQoL in favour of the ED and SM intervention. When multiple disability or HRQoL measures were reported, we used the trial's primary outcome. If the primary outcome was not specified by the authors, we selected the validated measure that was most reported across the included studies that were to be pooled in this specific meta‐analysis. Heterogeneity was evaluated using Cochrane's Q test and I^2^ (percentage of total variation due to between‐study heterogeneity) using Review Manager. Effect sizes of SMDs were interpreted as follows: < 0.2 was considered trivial, 0.2 to 0.49 small, 0.5 to 0.79 moderate and ≥ 0.8 large [30].

### 2.6 Certainty of Evidence and GRADE Appraisal

The Grading of Recommendations, Assessment, Development, and Evaluation (GRADE) framework was used to appraise the certainty of evidence and to formulate recommendations [31]. Levels of evidence were downgraded for serious risk of bias, unless risks of bias were solely associated with personnel and participants blinding, due to the nature of interventions being assessed in the current review. Levels of evidence were also downgraded for serious imprecision related to the magnitude of the CIs and for serious inconsistency based on heterogeneity and I^2^ statistics. Certainty levels were interpreted as follows:

- High certainty: Very confident the true effect lies close to calculated estimates.
- Moderate certainty: Moderately confident in the effect estimates. The true effect is likely to be close to the estimate, but there is still a possibility that it may differ substantially.
- Low certainty: Confidence in the effect estimates is limited. The true effect may be substantially different from the calculated estimate.
- Very low certainty: Very little confidence in the effect estimates. The true effect is likely to be substantially different from the calculated estimate.

## 3 Results

### 3.1 Study Selection

**FIGURE 1:**
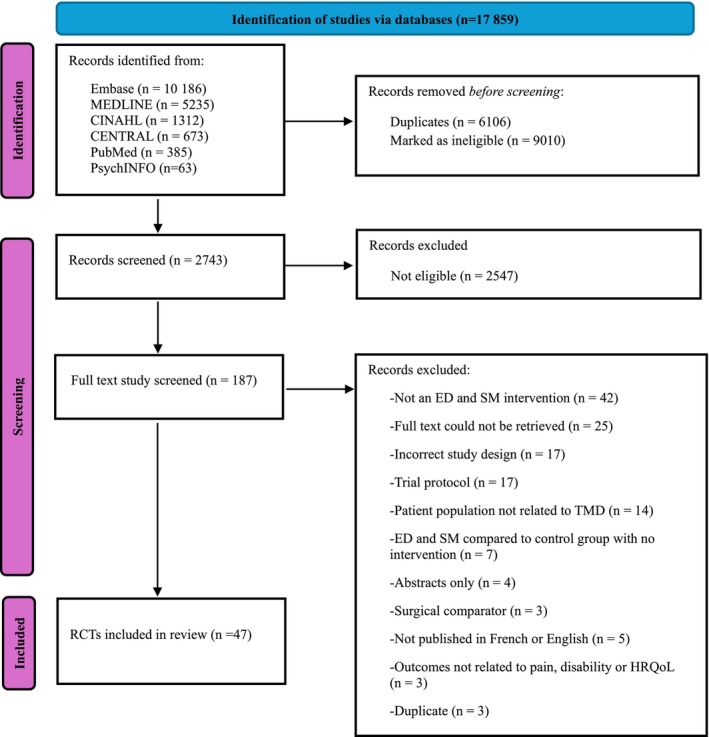
PRISMA flow diagram for the literature search.

The initial search found 17 859 records. After removing 6106 duplicates and 9010 records automatically marked as nonRCTs by Covidence, 2743 records were screened based on titles and abstracts. Of these, 187 studies were reviewed in full text. Forty‐seven studies met the eligibility criteria and were included in the systematic review, whereas 140 studies were excluded based on full‐text review (See File S2 for details on reasons for exclusion). The PRISMA flow diagram is presented in (Figure 1).

### 3.2 Characteristics of the Included Studies

#### 3.2.1 Participants

The 47 eligible trials included a total of 3238 participants, with women representing 76.8% of the overall sample. Sample sizes ranged from 19 to 186 participants (mean age: 34.4 ± 7.3 years). Twenty‐two trials included a sample population with mixed TMD diagnostic categories (myofascial pain, disc displacement, joint disorders). Nineteen studies included only participants with a diagnosis of myofascial pain. Four RCTs included participants with disc displacement with reduction [32, 33, 34, 35]. One study enrolled patients with disc displacement without reduction [36]. Fifteen RCTs included participants with persistent pain (3 months or more). Trials were conducted between 2000 and 2025, most of them occurring in university clinics (62%), with additional studies carried out in hospitals (11%) and private clinics (6%) (See File S3).

#### 3.2.2 Interventions

Over half of the trials involved dentists and dental hygienists (*n* = 21) or physiotherapists (*n* = 12) as care providers. ED and SM (*n* = 34) were most often combined with interventions such as occlusal splints, unsupervised home exercise programs or manual therapy (e.g., myofascial release, TMJ or cervical mobilizations) compared to ED and SM alone [4, 34, 35, 36, 37, 38, 39, 40, 41, 42, 43, 44, 45, 46, 47, 48, 49, 50, 51, 52, 53, 54, 55, 56, 57, 58, 59, 60, 61, 62, 63, 64, 65, 66]. In other trials (*n* = 15), ED and SM were compared to standalone treatments like splints, electrotherapy, or multimodal approaches [32, 33, 39, 46, 48, 53, 67, 68, 69, 70, 71, 72, 73, 74, 75]. Two trials also compared ED and SM added to other treatments versus those treatments alone [76, 77]. Intervention details are provided in (File S3 and S4). Components and delivery methods of ED and SM are outlined in Table 1 [25]. SM most commonly included unsupervised exercises (e.g., range of motion, strengthening or motor control), often based on Carlsson & Magnusson [78], or Rocabado's 6 × 6 exercise regime [79], though typically without a clear rationale. ED components focused primarily on behavioural modification to reduce masticatory overuse, but also addressed biomedical foundations of TMD, lifestyle and psychosocial factors, and posture education (e.g., whole body and tongue, mandible rest position). About half of these recommendations were explicitly sourced and when cited, prior trials were referenced (*n* = 24). Instructions for posture were generally vague.

**TABLE 1:** Description of education and self‐management components and delivery methods of rehabilitation programs from included RCTs.

1 Education and self‐management components	Number of programs	Relative frequency for all components (%) (*n* = 117)
1.1 Biomedical foundations of TMD^a^ (anatomy, physiology, aetiology, diagnostic, prognosis, pain physiology)	20	17
1.2 Overuse of the masticatory system modification (parafunctions, excessive movement, diet, avoid clenching)	28	24
1.3 Posture education (body, tongue and mandible)	14	12
1.4 Lifestyle and psychosocial education (sleep, physical activity, psychosocial components)	19	16
1.5 Self‐management instructions (exercise, thermotherapy and additional support)	36	31
Total	117	
**2. Delivery methods**	**Number of occurrences**	**Relative frequency for all methods (%) (*n* = 78)**
2.1 Contextual example	0	0
2.2 Education session	43	55
2.3 Group interaction	0	0
2.4 Personalized sessions	3	4
2.5 Self‐reflection/application	0	0
2.6 Video‐based learning	1	1
2.7 Written materials	27	35
2.8 Not reported	4	5
Total	78	

^a^ TMD, Temporomandibular disorders.

ED and SM were delivered mostly via brief one‐on‐one sessions (*n* = 43, 91%), with leaflets or visual aids provided in 57% (*n* = 27) of trials. Programs were usually standardized; only 6% (*n* = 3) individualized ED and SM to patient complaints [53, 60, 62]. Session duration was rarely specified (*n* = 8) [42, 46, 47, 54, 71, 72, 76, 77]. When reported, it ranged from 15 to 75 min. Multiple in‐person sessions were uncommon (*n* = 16), and follow‐ups by phone were reported in only three trials [37, 47, 62]. See File S5.

#### 3.2.3 Outcome Measures

Pain intensity was a primary outcome measure in 45 of the included studies. See File S3. Its measurements mostly involved patients' self‐reported pain in a resting posture using a VAS or a NRS. Function was measured using validated self‐reported questionnaires, such as the Jaw Functional Limitation Scale (JFLS‐8 or JFLS−20, *n* = 6) or the Mandibular Function Impairment Questionnaire (*n* = 2) or using a patients' perceived functional status on a 0–10, 0–100 scale or a five‐point Likert scale (0–4) when asking for the level of limitation during jaw movements. HRQoL was measured either with the Oral‐Health Impact Profile (*n* = 4) or the World Health Organization Quality of Life, brief version (*n* = 2). Thirty‐five RCTs evaluated short‐term (1–6 weeks), 22 medium‐term (7–26 weeks) and five long‐term effects (27–52 weeks).

### 3.3 Risk of Bias

Among the 47 included studies, no single RCT was considered at low risk of bias by the RoB 1, 3 RCTs presented unclear risk of bias, and 44 RCTs were considered at high risk of bias. See File S6. Three RCTs presented selection bias due to inadequate processes in the generation and concealment of the group allocation [33, 37, 38]. For example, two RCTs randomized participants based on their order of arrival in the trial [37, 38], or group allocation was made through direct contact, without concealment [33]. According to the nature of the interventions, all but four RCTs presented a high risk of performance bias due to a lack of blinding among therapists administering the intervention and/or among participants. More specifically, two RCTs were considered as presenting a low risk of performance bias since double‐blinding was mentioned, and interventions were similar enough to be undetected by participants with clear strategies to ensure blinding [72, 76]. Two RCTs were considered with unclear performance bias because those details were not explicitly reported [45, 55]. Six RCTs showed a detection bias due to knowledge of the allocated interventions by outcome assessor [32, 37, 41, 59, 67, 73]. Sixteen RCTs presented attrition bias due to the amount, nature, or handling of incomplete outcome data or proper statistical analyses. In the case of five RCTs, concerns arose for selective reporting [35, 39, 46, 69, 76]. Two RCTs performed group exchange of participants [52, 58]. One study excluded participants after randomization [62]. Inadequate sample size and low statistical power were present in two studies [72, 77].

### 3.4 The Efficacy of ED and SM Compared to Other Interventions

**FIGURE 2:**
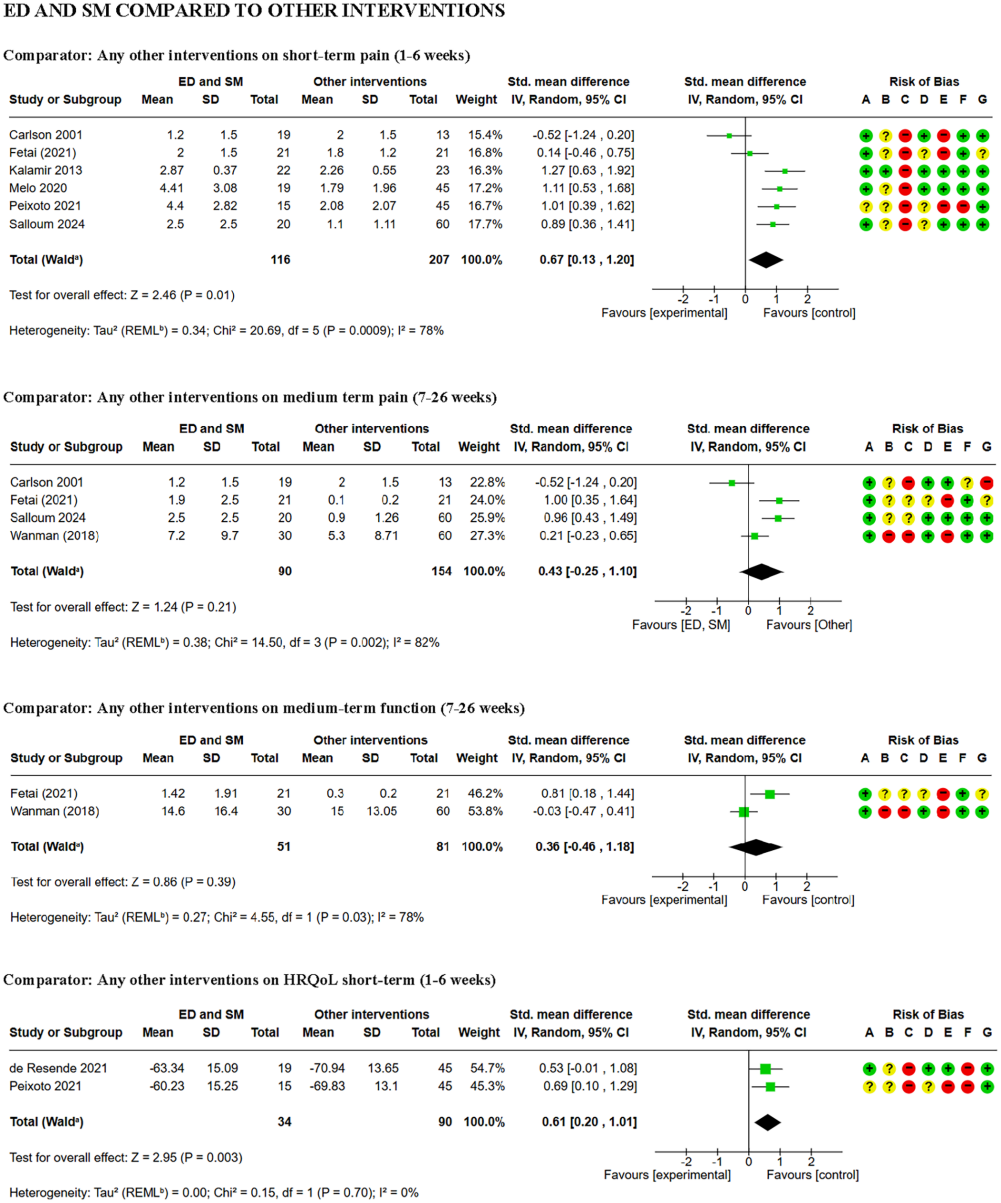
Meta‐analyses comparing ED and SM to other interventions.

Fifteen RCTs assessed the efficacy of ED and SM compared to other interventions for reduction of pain, functional limitations and improvement of HRQoL. Of those, eight RCTs were pooled into meta‐analyses. Overall, interventions other than ED and SM showed a significant effect compared to ED and SM for short‐term pain reduction (SMD = 0.79, 95% CI: 0.27–1.32, I^2^ = 78%, 7 studies, 359 patients). But, for medium‐term pain reductions (SMD = 0.43, 95% CI: −0.25–1.10, I^2^ = 82%, 4 studies, 144 patients) and medium‐term function improvements (SMD = 0.36, 95% CI: −0.46–1.18, I^2^ = 78%, 2 studies, 132 patients), other interventions were not significantly superior to ED and SM. For short‐term HRQoL improvements, other interventions were significantly superior to ED and SM alone (SMD = 0.61, 95% CI: 0.20–1.01, I^2^ = 0%, 2 studies, 124 patients). See Figure 2 and File S7. Seven trials, all with a high risk of bias, were not included in meta‐analyses. Three RCTs assessing short‐term pain found that ED and SM interventions, splints and electrotherapy, all significantly reduced pain [39, 67, 70]. Between‐group comparisons favoured splints and electrotherapy over ED and SM in two studies [39, 70], while one found no significant difference [67]. Six high‐risk of bias RCTs evaluated medium‐term pain, where four trials (ED, SM, splints) showed significant within‐group reductions of pain [32, 39, 67, 72]. Only one trial reported significant between‐group effects in favour of splints [73], and another in favour of ED and SM [71], while others did not report significant between‐group differences. Two high risk of bias RCTs assessed medium‐term function improvements and showed similar improvements for both groups, ED and SM and use of splints [72, 73]. See Files S3 and S6.

### 3.5 The Addition of ED and SM to Another Intervention Compared to the Intervention Alone

**FIGURE 3:**
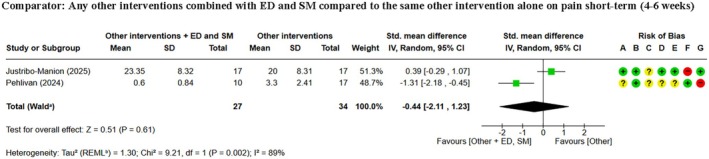
Meta‐analyses comparing ED and SM combined with any other interventions compared to ED and SM alone.

Only two RCTs assessed the effectiveness of the addition of ED and SM to another interventions that included manual therapy or a multimodal treatment (i.e., electrotherapy modalities and supervised exercises) [76, 77]. For short‐term pain reduction, this combination showed no significant benefit compared with the intervention alone (SMD = −0.44, 95% CI: −2.11–1.23, I^2^ = 89%, 2 studies, 61 patients). See Figure 3.

### 3.6 The Efficacy of ED and SM Combined to Another Intervention Compared to ED and SM Alone

**FIGURE 4:**
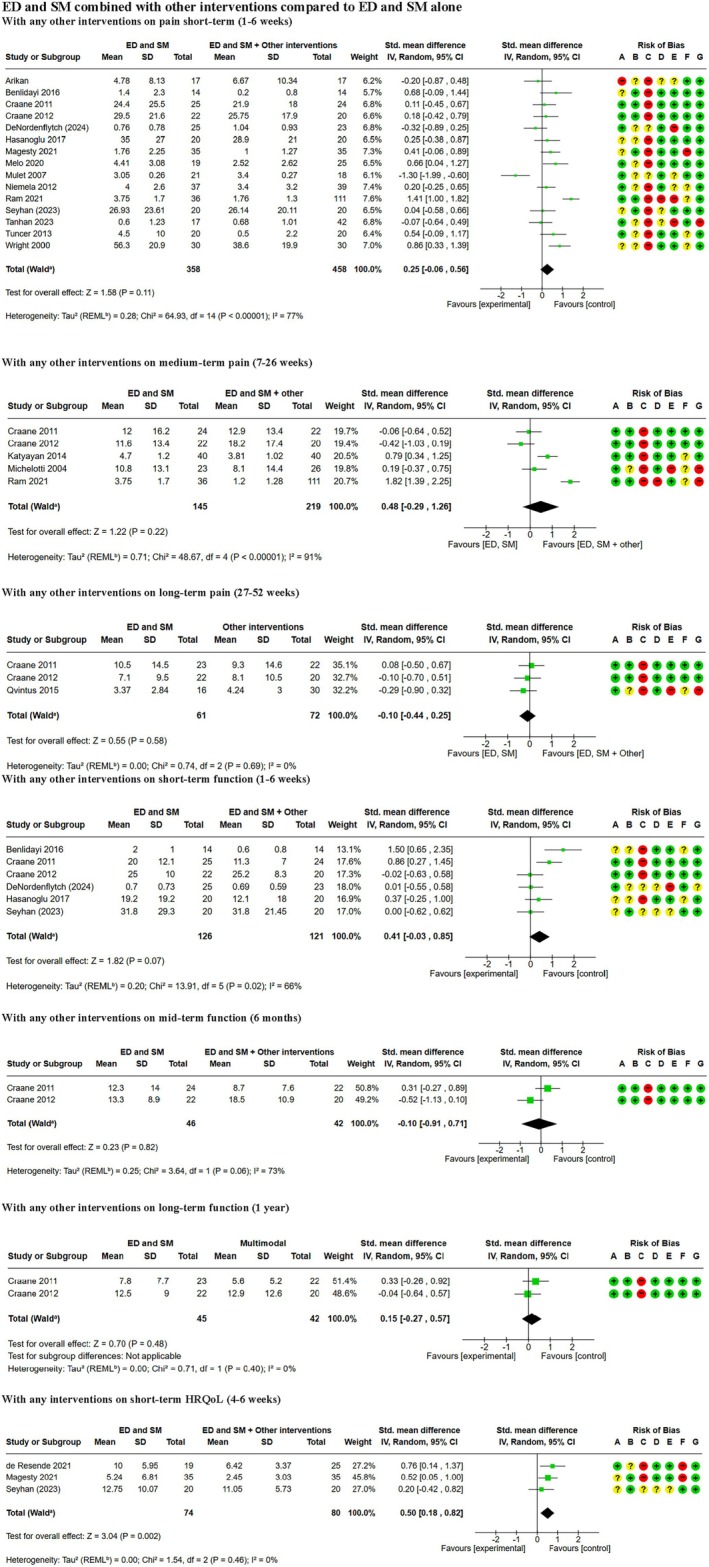
Meta‐analyses comparing ED and SM combined with any other interventions compared to ED and SM alone.

Thirty‐four high‐risk‐of‐bias RCTs compared ED and SM combined with another intervention to ED and SM alone on the reduction of pain, functional limitations and improvement of HRQoL. Nineteen RCTs were pooled into meta‐analyses. For all outcomes and follow‐ups, besides those of short‐term HRQoL improvements, the combination of ED and SM with another intervention was not significantly superior to ED and SM alone. The degree of heterogeneity of those results was significant (*p* < 0.05), except for long‐term pain reductions and long‐term function improvements, where no heterogeneity was observed (I^2^ = 0%). See Figure 4 and File S7. Only for short‐term HRQoL improvements was the combination of another intervention with ED and SM superior to ED and SM alone (SMD = 0.50, 95% CI: 0.18–0.82, I^2^ = 0%, 3 studies, 154 patients).

Fourteen trials were not pooled into this meta‐analysis. Eight trials with a high risk of bias assessed short‐term pain and found that between‐group comparisons favoured combining ED and SM with other modalities such as splints, electrotherapy, manual therapy and unsupervised exercise programs in six studies [34, 39, 48, 49, 61, 65], while two found no significant differences between groups [37, 41]. Five RCTs with a high risk of bias evaluated medium‐term pain [4, 39, 43, 47, 56], where three studies favoured adding splints to ED and SM compared to ED and SM alone, while one reported superior outcomes for ED and SM alone [47], and one found no differences between approaches [56]. Two studies with high risk of bias assessed long‐term pain and found either no between‐group differences [63] or better outcomes for ED and SM alone [47]. Two studies with a high risk of bias assessed short‐term functional improvements: the combination of ED and SM with dry needling or multimodal treatment did not reach significant between‐group differences [37, 41]. One RCT [63] examined long‐term function and found significant improvements in all groups without inter‐group differences. Another study [52] assessed medium‐ and long‐term HRQoL and found improvements in all groups, again with no significant differences between interventions. See Files S3 and S6.

### 3.7 Certainty of Evidence

According to the GRADE assessment, the certainty of evidence was rated as very low for most patient‐reported outcomes and follow‐up periods, except for two outcomes with low certainty. Downgrades in the certainty of evidence were primarily due to risk of bias and imprecision of confidence intervals. See Table 2 for the summary of findings according to GRADE.

**TABLE 2:** Summary of Findings according to GRADE for ED and SM and other interventions for TMDs.

	Outcome and follow‐up	Patients (studies), N	SMD (95% CI)	Certainty	Conclusions
ED and SM alone compared to other interventions	Short‐term pain	323 (6 RCTs)	0.67 (0.13 to 1.2)	⨁◯◯◯ Very low^a^, ^b^, ^c^	The evidence is very uncertain. The confidence intervals are large but include small to large effects that favour other interventions compared to ED and SM alone on short‐term pain.
	Medium‐term pain	244 (4 RCTs)	0.43 (−0.25 to 1.1)	⨁◯◯◯ Very low^a^, ^b^, ^c^	The evidence is very uncertain. The confidence intervals are large and include small to large effects that may favour either intervention.
	Medium‐term function	132 (2 RCTs)	0.36 (−0.46 to 1.18)	⨁◯◯◯ Very low^a^, ^b^, ^c^	The evidence is very uncertain. The confidence intervals are large and include moderate to large effects that may favour either intervention.
	Short‐term HRQoL	124 (2 RCTs)	0.61 (0.2 to 1.01)	⨁◯◯◯ Very low^a^, ^c^	The evidence is very uncertain. The confidence intervals are large but include small to large effects that favour other interventions on short‐term HRQoL improvements.
ED and SM combined to other interventions compared to the interventions alone	Short‐term pain	61 (2 RCTs)	−0.44 (−2.11 to 1.23)	⨁◯◯◯ Very low^a^, ^b^, ^c^	The evidence is very uncertain. The confidence intervals are large and include large effects that may favour either group.
ED and SM combined with other interventions compared to ED and SM alone	Short‐term pain	816 (15 RCTs)	0.25 (−0.06 to 0.56)	⨁◯◯◯ Very low^a^, ^b^, ^c^	The evidence is very uncertain. The confidence intervals are large and include trivial to moderate effects that may favour either group.
	Medium‐term pain	364 (5 RCTs)	0.48 (−0.29 to 1.26)	⨁◯◯◯ Very low^a^, ^d^, ^e^	The evidence is very uncertain. The confidence intervals are large and include moderate to large effects that may favour either group.
	Long‐term pain	133 (3 RCTs)	−0.1 (−0.44 to 0.25)	⨁⨁◯◯ Low^a^, ^e^	The evidence suggests that ED and SM alone, when compared to ED and SM combined with other interventions, may result in a small to moderate reduction in long‐term pain that could favour either group.
	Short‐term function	247 (6 RCTs)	0.41 (−0.03 to 0.85)	⨁◯◯◯ Very low^a^, ^b^, ^c^	The evidence is very uncertain. The confidence intervals are large and include small to large effects that may favour either group.
	Medium‐term function	88 (2 RCTs)	−0.1 (−0.91 to 0.71)	⨁◯◯◯ Very low^b^, ^e^	The evidence is very uncertain. The confidence intervals are large and include small to large effects that may favour either group.
	Long‐term function	87 (2 RCTs)	0.15 (−0.27 to 0.57)	⨁⨁◯◯ Low^e^	The evidence suggests that the addition of another intervention to ED and SM, when compared to ED and SM alone, may result in a small to moderate improvement in long‐term function that could favour either group.
	Short‐term HRQoL	154 (3 RCTs)	0.5 (0.18 to 0.82)	⨁◯◯◯ Very low^c^, ^f^	The evidence is very uncertain. The confidence intervals are large and include small to large effects that may favour other intervention combined with ED and SM compared to ED and SM alone on short‐term HRQoL.

Abbreviations: CI, confidence interval; ED and SM, Education and self‐management; HRQoL, Health‐related quality of life; N, Number of patients; RCT, Randomised controlled trial; SMD, standardised mean difference.

^a^ Downgraded one level for some concerns with items of RoB‐1.

^b^ Downgraded one level for some inconsistent results.

^c^ Downgraded one level for imprecise confidence interval.

^d^ Downgraded two levels for inconsistency.

^e^ Downgraded two levels for imprecise confidence interval.

^f^ Downgraded two levels for risk of bias with one item or more, or some concerns with multiple items of RoB‐1.

## 4 Discussion

This systematic review evaluated the efficacy of ED and SM interventions compared to other non‐surgical treatments in adults with TMDs. While some short‐term benefits may favour other non‐surgical interventions, particularly for pain and HRQoL, the overall certainty of the evidence remains low to very low, with no strong or consistent indication that ED and SM are either superior or inferior to commonly used interventions such as occlusal splints, manual therapy, supervised therapeutic exercises or multimodal approaches. ED and SM interventions were generally delivered as standardized programs and often served as control conditions lacking clear grounding in best practice or based on clinical guidelines. This, combined with the limited number of high‐quality trials, substantial heterogeneity in intervention content and study populations, may have limited their ability to demonstrate benefits for TMD care.

Based on very low‐certainty evidence, non‐surgical interventions such as splints, manual therapy, electrotherapy or a multimodal approach may be clinically superior to ED and SM alone for short‐term pain reduction. Similarly, very low‐certainty evidence suggests that non‐surgical interventions such as splints, supervised and unsupervised exercises or multimodal approaches, either alone or in combination with ED and SM, may yield moderate clinically relevant improvement for HRQoL in the short term compared to ED and SM alone. Aside from these two specific findings, low‐ to very low‐certainty evidence suggests that the effects of ED and SM alone are comparable to those of other non‐surgical interventions, whether delivered alone or in combination with ED and SM, with no clear superiority of one approach over the other. These results are consistent with previous systematic reviews [24, 25, 26, 27, 28, 29, 30, 31, 32, 33, 34, 35, 36, 37, 38, 39, 40, 41, 42, 43, 44, 45, 46, 47, 48, 49, 50, 51, 52, 53, 54, 55, 56, 57, 58, 59, 60, 61, 62, 63, 64, 65, 66, 67, 68, 69, 70, 71, 72, 73, 74, 75, 76, 77, 78, 79], which also identified ED and SM as potentially beneficial, low‐cost first‐line treatment options for TMD care offering comparable clinically relevant effects to more complex and resource‐intensive approaches.

To our knowledge, this is the first systematic review with meta‐analyses to assess the effectiveness of ED and SM compared with other non‐surgical interventions in TMD care. Pooling evidence across trials proved challenging due to substantial methodological variability, including different outcome measures, reporting and follow‐up time points. As a result, several studies could not be included in the quantitative syntheses, but qualitative analyses were also performed, which did not change our conclusions based on the meta‐analyses. Furthermore, sensitivity analyses were conducted according to risk of bias assessment and did not change our main conclusions. In the current review, most included trials presented a significant risk of bias [22] because of inadequate randomization or allocation concealment, lack of blinded outcome assessments and insufficient statistical power to detect meaningful effects. Beyond these methodological concerns, another notable limitation of the current evidence is the short duration of follow‐up in most trials. While the majority assessed outcomes over 1 to 6 weeks, only a small number examined medium‐ to long‐term effects (7 to 52 weeks). This short‐term focus limits our understanding of the sustained potential impact of ED and SM on pain, function and HRQoL which is important to understand therapeutic effect for such recurrent and chronic conditions including TMDs [80].

ED and SM interventions remain a central component of multimodal TMD management in clinical practice, typically combining therapeutic education, behavioural strategies, healthy lifestyle promotion and self‐guided exercises [20, 24, 25, 80, 81]. When such interventions are patient‐centered, individualized and rooted in behavioural change, they may support health literacy, empower patients to actively manage symptoms, and potentially reduce healthcare utilization and costs [82, 83]. However, while some protocols in included trials referenced existing frameworks or prior research, most were developed independently by research teams, generally employing standardized, generic programs delivered as a control condition with limited clarity regarding their theoretical foundations, delivery methods, or alignment with evidence‐based recommendations. This lack of personalization contrasts sharply with current recommendations for patient‐centered care in musculoskeletal and TMD care and may partly account for the inconsistent findings observed across our review and meta‐analyses.

## 5 Strengths and Limitations

Strengths of this systematic review and meta‐analysis are its rigorous alignment with well‐designed frameworks related to TMD diagnostic criteria for the inclusion of trials and with PRISMA and Cochrane Collaboration guidelines [22, 25, 26, 27, 28, 29, 30, 31, 32, 33, 34, 35, 36, 37, 38, 39, 40, 41, 42, 43, 44, 45, 46, 47, 48, 49, 50, 51, 52, 53, 54, 55, 56, 57, 58, 59, 60, 61, 62, 63, 64, 65, 66, 67, 68, 69, 70, 71, 72, 73, 74, 75, 76, 77, 78, 79, 80, 81, 82, 83, 84]. The comprehensive search strategy across six databases, inclusion of both qualitative and quantitative syntheses, and the use of GRADE to assess certainty of evidence further enhance the robustness of findings. Nevertheless, some limitations must be acknowledged. Although we included a large number of trials, our ability to perform subgroup analyses, either by specific diagnoses of TMDs or by different ED and SM delivery methods was not possible because of the variability in designs and study population represented in the trials. Moreover, as most trials included participants with myofascial TMD, caution is warranted in generalizing findings to other diagnostic subgroups, such as disc displacement or arthrogenic disorders. Another limitation concerns the language restrictions applied in this review. The search strategy was conducted only in English and French, which are the languages spoken and understood by the research team. As a result, relevant studies published in other languages may not have been captured. Additionally, the risk of bias assessment was performed using the original RoB 1. While widely used, RoB 1 provides less granular assessment criteria compared to the updated RoB‐2 tool, which may impact the precision with which risk of bias can be interpreted.

Despite these limitations, this review offers a meaningful contribution to understanding the role of ED and SM TMD care. It highlights both the potential value of these interventions and the urgent need for high‐quality, theory‐informed trials that explore their effectiveness across diverse TMD populations and clinical settings.

## 6 Conclusion

Overall, the certainty of evidence supporting the use of education and self‐management for adults with temporomandibular disorders remains very low to low across all outcomes and time points. While some short‐term clinically relevant benefits may favour other non‐surgical interventions for pain and HRQoL, there is no consistent evidence that ED and SM is inferior or superior to other non‐surgical treatments. Similarly, combining ED and SM with other interventions did not consistently improve outcomes compared to ED and SM alone. The wide confidence intervals, methodological limitations and high risk of bias across studies indicate that more rigorous, high‐quality trials are needed to clarify the role of ED and SM interventions in TMD care, especially regarding medium‐ and long‐term improvements in pain, function and HRQoL. Furthermore, more comprehensive, evidence‐based and patient‐centered ED and SM interventions should be tested for adults with TMDs.

## Conflicts of Interest

The authors declare no conflicts of interest.

## Supporting information


**File S1:** Search strategy.

**File S2:** Reasons for full text exclusion.

**File S3:** Characteristics of the included studies (*n* = 47).

**File S4:** Details on the ED and SM interventions.

**File S5:** Table of the descriptive parameters of ED and SM.

**File S6:** Cochrane Risk of Bias v1.

**File S7:** Sensitivity analyses.

## Data Availability

The data that support the findings of this study are available from the corresponding author upon reasonable request.
