# Supplementary material for: Patient Education and Self‐Management in Adults With Temporomandibular Disorders: Results From a Systematic Review With Meta‐Analysis
Source: J Oral Rehabil. 2026 Mar 19;53(7):1394–408. doi: 10.1111/joor.70187 (PMC13261784; doi:10.1111/joor.70187)
Supplement: Supplementary file 1 — File S1: Search strategy. [file JOOR-53-1394-s003.docx]

| **Concepts** | **Concept 1**  Temporomandibular disorders | **Concept 2**  Education |
| --- | --- | --- |
| **Keywords** | Temporomandibular joint  Temporomandibular disorder  Temporomandibular dysfunction  Temporomandibular disease  Temporomandibular syndrome  Temporomandibular pain  Jaw disease  Jaw joint  Facial pain  Bruxomania  Bruxism  Costen syndrome | Counselling  Education  Self-management  Self-care  Self treatment  Self nurturance  Patient education  Patient knowledge  Health knowledge  Health education  Home physical therapy  Exercise  instructions |
| **Search equations** | (Jaw adj3 (disease* OR joint*)) or ((mandib*) adj3 (articulation* OR joint* OR disorder* OR syndrome* OR disease* OR dysfunction* OR pain OR painful)) OR costen syndrome OR bruxism OR bruxomania OR "fac?ial pain" | Counsel?ing OR (self NEAR/3 (management OR treatment OR care OR nurturance)) OR ((Education OR knowledge) NEAR/2 (patient* OR health)) OR “home physical therap*” OR exercise* |
| **MeSH terms** | Exp temporomandibular joint/  Exp temporomandibular joint disc/  Exp temporomandibular joint disorder/  Exp temporomandibular ankylosis/  Exp jaw disease/  Temporomandibular joint disorders/  Temporomandibular joint dysfunction syndrome/  Bruxism/  Sleep bruxism/  Jaw/  Exp facial pain/  Exp maloclussion/  Exp craniomandibular disorders/  Facial muscles/  Exp masticatory muscles/ | Exp counseling/  Exp self-care/  Exp patient education/  Exp self-care education/ |

**Supplementary File S1**. Search Strategy
