# Supplementary material for: Patient Education and Self‐Management in Adults With Temporomandibular Disorders: Results From a Systematic Review With Meta‐Analysis
Source: J Oral Rehabil. 2026 Mar 19;53(7):1394–408. doi: 10.1111/joor.70187 (PMC13261784; doi:10.1111/joor.70187)
Supplement: Supplementary file 2 — File S2: Reasons for full text exclusion. [file JOOR-53-1394-s007.docx]

**Supplementary File S2.** Full text not found (n = 25) and main reasons for exclusion at full text level (n = 115)

|  | **Study** |
| --- | --- |
| Full text not found  (n=25) | Unknown authors, unknown authors (2014), unknown authors (2020), unknown authors (2018), Ates K. (2019), unknown authors (2024), unknown authors (2018), unknown authors (2022), Chun-Hua Y. (2020), Costa Y. (2021), Deskapan A. (2011), de Negreiros WA. (2025), Goldstein L.B (1985), Magnusson T. (1999), Morrone L. (1991), Pehlivan Tekin G. (2018), Alagia Thiruvevenkadam I. (2021), unknown authors (2023), unknown authors (2019), unknown authors (2023), unknown authors (2021), unknown authors (2023), unknown authors (2012), unknown authors (2018), unknown authors (2023), |
| **Main reason for exclusion** | **Study (n=115)** |
| Not an (isolated) ED and SM intervention (n=42) | Maluf SA. (2010), Haketa T. (2010), Tariq M. (2023), De Laat A. (2003), dos Santos Aguiar A. (2023), Alencar FG. (2014), Barbosa MA. (2019), Baykan O. (2022), Centazo N. (2024), Cho YK. (2025), de Oliveira-Souza A.I.S (2024), Elfekey ESA. (2022), Ficnar T. (2013), Giannakopoulos N.N. (2016), Garrigos-Pedron M. (2018), Grace E.G. (2002), Herman C. (2002), Jo JH (2021), Kalamir A. (2010), Kanungo B. (2020), Ke X. (2022), Kraaijenga S. (2014), Kulekcioglu S. (2003), Lee L-S. (2023), Maluf SA. (2011), McArdle W.D. (1984), Minakuchi H. (2001), Miotto E. (2021), Moleirinho-Alves P.M.M (2021), Moleirinho-Alves P.M.M (2021), Nagata K. (2015), Nascimento M.-M. (2013), De la Torre Canales G. (2019), Regis RR. (2015), Reynolds, B. (2020), Reynolds, B. (2020), Ritenbaugh, C. (2012), Romeo A. (2024), Sahin D. (2021), Turk DC. (1996), Turner J.A. (2005), Zhu R. (2024) |
| Incorrect study design (n=17) | Kalamir A. (2010), Brignardello-Petersen R. (2019), Cros P. (1997), Elimairi I. (2017), John MT (2007), Kurt H. (2011), Manfredini D. (2018), McDonnell MK (2005), Falcão Carvalho Porto de Freitas R. (2014), Nicolakis P. (2001), Nicolakis P. (2002), Österlund C. (2019), Sarfraz S. (2023), Serrano-Hernanz, G. (2023), von Piekartz H. (2024), Wilkoff R. (1984), Zakrzewska J.M. (2012) |
| Trial protocol (n=17) | Salinas Aguilar J. (2023), Ergezen G. (2023), Spavieri J. (2024), Castro-Sánchez AM. (2015), Ortigosa Cunha C. (2020), Bilgen B. (2024), Mardomingo Medialdea H. (2024), Ortiz Moreno MT. (2024), Campos A. (2022), Dantony F. (2024), Tuncer A. (2023), Gençosmanoğlu H. (2023), Gençosmanoğlu H. (2023), dos Santos Aguiar A. (2019), Kuru Colak T. (2022), Dumont Flecha O. (2020), Boonprakob Y. (2018) |
| Patient population not related to adults with TMD (n = 14) | Coşkun Karataş S. (2025), Çalışgan E. (2018), Santos Miotto Amorim CS. (2014), Atay F. (2023), Mehmet Micoogullari (2023), Kadıoğlu MB. (2024), Acar B. (2012), Debre E. (2020), Delgado de la Serna P. (2020), Halmova K (2017), Kumar S.S. (2019), Tsolka P. (1992), Turcio KHL. (2011), Ucar I. (2022) |
| ED and SM compared to control group with no intervention (n=7) | Atilgan E. (2024), Kalamir A. (2012), Kleinrok M. (1972), Lopes de Castro Bastos T. (2018), Nicolakis P. (2001), Yoshida H. (2011), Yuasa H. (2001) |
| Abstracts (n=4) | Michelotti A. (2002), Lucas C. (2018), Moleirinho-Alves P. (2021), Santos Miotto De Amorim C. (2015) |
| Surgical comparator (n=3) | De Almeida A.M. (2023), Ferreira D. (2024), Kleinrok M. (1972) |
| Not published in French or English (n=5) | Acar B. (2012), Michelotti A (2000), Silant'eva E.N. (2010), van der Glas HW. (2000), Yu C.-H. (2016) |
| Outcomes not related to pain, disability or HRQoL (n = 3) | Chattrattrai T. (2024), Chattrattrai T. (2024), Oliveira S.S.I. (2019) |
| Duplicate (n = 3) | Aksu O. (2019), Kokkola O. (2018), Ram HK. (2021) |
