# Supplementary material for: Patient Education and Self‐Management in Adults With Temporomandibular Disorders: Results From a Systematic Review With Meta‐Analysis
Source: J Oral Rehabil. 2026 Mar 19;53(7):1394–408. doi: 10.1111/joor.70187 (PMC13261784; doi:10.1111/joor.70187)
Supplement: Supplementary file 3 — File S3: Characteristics of the included studies (n = 47). [file JOOR-53-1394-s001.docx]

| **Context** | | | **Participants** | | | | **Interventions** | | | **Measurements** | | **Results** |
| --- | --- | --- | --- | --- | --- | --- | --- | --- | --- | --- | --- | --- |
| **Authors, year** | **Country** | **Care setting(s)** | **n** | **Female %** | **Mean Age (SD)** | **TMD Diagnostic categories** | **Providers** | **Treatment arms** | **Themes; delivery method; parameters of ED and SM** | **Outcome** | **Follow-up** |  |
| Aksu et al., 2019 | Turkey | Hospital | 21  20 | NS | NS | Myofascial pain Chronic | Physiatrists | G1: ED and SM  G2: ED and SM + Dry needling | Self-massage, diet, relaxation, exercises (strength, coordination, stretch); Print-out; 1 session + 3 phone follow-ups) | Pain Fct | 4 weeks | At 4 weeks, both groups demonstrated a significant reduction of pain and Fct (p<0.001) after treatment;  There was no difference between the groups in pain (p = 0.557) and Fct after treatment. |
| Arikan et al., 2025 ^c^ | Turkey | University clinic | 17  17 | 64.7 %  64.7 % | 33.6 (8.1)  27.8 (7.2) | Mixed Chronic | Physiotherapists | G1: ED and SM  G2: ED and SM + HVES | Posture, diet, parafunctions, symmetrical usage, excessive movements, breathing, relaxation, exercises (mobility, strength, stretch, coordination, relaxation); Oral instructions; 1 session | Pain | 4 weeks | At 4 weeks, both groups demonstrated a significant reduction of pain (p=0.001) after treatment  No significant difference between the groups in pain intensity (p=0.679) after treatment. |
| Benli et al., 2024 | Turkey | University clinic | 18  17  18  17  18 | 55.6 %  70.6 %  61.1 %  64.7 %  72.2 % | 32.6 (5.9)  31.3 (7.6)  28.9 (4.7)  35.3 (7.1)  28.8 (9.1) | Myofascial pain | Dentists | G1: ED and SM  G2: Earplug  G3: Splint  G4: Earplug + splint  G5: ED and SM + Earplug | Self-care exercises (stretch, coordination, relaxation); Oral instructions; 1 session | Pain | 4 weeks 3 months | All groups demonstrated a significant reduction in pain at all time points;  G4 and G5 showed significantly greater reduction in pain than other groups (p<0.05). |
| Benlidayi et al., 2016 ^c^ | Turkey | NS | 14 14 | 78.6 %  92.9 % | 31.1 (10.1)  31.6 (11.5) | Mixed | Physician | G1: ED and SM  G2: ED and SM + Kinesio taping | Lifestyle counseling, exercises (mobility, strength); Written instructions; 1 session | Pain Fct | 6 weeks | At 6 weeks, only G2 achieves a significant reduction in pain (p=0.011) and improvement in Fct (p=0.001);  G2 showed a significantly greater reduction in pain (p=0.046) and improvement in Fct (p=0.00) than G1 at 6 weeks. |
| Brandão et al., 2022 | Brazil | University clinic | 8 11 | 100 %  73.0 % | 36.7 (11.2)  35.4 (10.4) | Mixed | NS | G1: ED and SM  G2: ED and SM + Multimodal | Diet, parafunctions, control excessive opening; Oral instructions, 1 session | Pain Fct | 4 weeks | At 4 weeks, G1 and G2 did not achieve any significant reduction in pain or improvement in Fct,  No significant differences between groups were found for pain or Fct. |
| Carlson et al., 2021 ^a^ | USA | National Medical Center | 23 21 | 77.3 % | 34.6 (NS) | Myofascial pain | Dentists | G1: ED and SM  G2: ED and SM + Multimodal | Pain education, etiology, parafunctions, sleep, relaxation, physical activity, diet, breathing, proprioceptive awareness; Oral instructions + hand-out; 2x 50 min sessions | Pain | 6 weeks  6 months | At 6 weeks, G1 and G2 achieved significant reductions in pain (p<0.05);  G1 showed a greater reduction in pain than G2 at 6 months (p<0.05). |
| Conti et al., 2012 | Brazil | University clinic | 14 21 16 | 100 %  80.9 %  87.5 % | 38.1 (NS) 38.1 (NS) 35.3 (NS) | Myofascial pain | Dentists | G1: ED and SM  G2: ED and SM + Splint  G3: ED and SM + Splint (NTI-tss) | Behavioral changes, relaxation, sleep, diet, thermotherapy, self-massage; print-out; 4 sessions | Pain | 2 weeks 6 weeks 3 months | At 3 months, all groups achieved significant reductions in pain (p<0.05); G2 showed a greater reduction in pain than G1 at 6 weeks. |
| Conti et al., 2015 | Brazil | Orofacial Pain Clinic | 20 20 20 | 96.7 % | 46.0 (NS) 38.4 (NS) 38.4 (NS) | Mixed | Dentists | G1: ED and SM  G2: ED and SM + Splint  G3: ED and SM + Splint (NTI-tss) | Relaxation, sleep, diet, thermotherapy; Oral instructions; 4 sessions | Pain | 2 weeks 6 weeks 3 months | At 3 months, all groups achieved significant reductions in pain (p<0.05);  G3 showed greater reduction in pain than G1 at 6 weeks (p=0.003) and 3 months (p=0.01). |
| Craane et al., 2011 ^c^ | Belgium | University clinic | 27  26 | 74.1 %  76.9 % | 42.9 (15.1) 36.3 (15.3) | Myofascial Pain | Physiotherapists | G1: ED and SM  G2: ED and SM + Multimodal | Physiology, parafunctions, relaxation, excessive opening; Oreal and written instructions; 6 sessions | Pain Fct | 3 weeks 6 weeks 3 months 6 months 1 year | At 1 year, both groups achieved significant reductions in pain and Fct;  No significant differences between groups were found for pain (p=0.871) and Fct (0.209) at 1 year. |
| Craane et al., 2012 ^c^ | Belgium | University clinic | 26 23 | 92.3 %  100 % | 38.5 (15.1) 34.7 (14.0) | DDwR | Physiotherapists | G1: ED and SM  G2: ED and SM + Multimodal | Physiology, parafunctions, relaxation, excessive opening; Oral and written instructions; 6 sessions | Pain Fct | 3 weeks 6 weeks 3 months 6 months 1 year | At all follow-up time points, G1 and G2 achieved significant reductions in pain and improvements in Fct; No differences between groups were found for pain and Fct at all follow-up time-points (p>0.05). |
| DeNordenflytch et al,. 2024 ^c^ | Chile | University clinic | 25  23 | 73.0 % | 27.6 (5.9) 24.3 (4.2) | Myofascial pain | Dentists | G1: ED and SM  G2: ED and SM + Unsupervised exercises | Etiology, prognosis, behavioral changes, relaxation, sleep, diet, thermotherapy, social and aerobic activities, healthy living habits; Audio-visual material and verbal instructions; 4 sessions | Pain Fct | 2 weeks 6 weeks 10 weeks | Intra-group: Both groups demonstrated a significant reduction of pain at all time-points (p<0.001) and Fct (p=0.000) after treatment;  No significant differences between the groups for pain (p=0.809, 0.415, 0.275) and Fct (p=0.657, 0.671) after treatment. |
| de Resende et al., 2021 ^a,c^ | Brazil | University clinic | 19  24  21  25 | 80.9 % | 28 (9.3) | Mixed | Dentists | G1: ED and SM  G2: Splint  G3: Multimodal  G4: ED and SM + Splint | Etiology, parafunctions, awareness of clenching, excessive opening, physical activity, posture, sleep; Oral instructions; 2 x 30 min sessions | Pain  HRQoL | 4 weeks | At 4 weeks, all groups achieved significant reductions in pain and improvements in HRQoL (p<0.001);  No significant differences between any groups were found for pain and HRQoL (p>0.05). |
| Dworkin et al., 2002  RDC/TMD: Research Diagnostic Criteria for Temporomandibular Disorders, NS: Not specified, MT: Manual Therapy, NTI-tss: Nociceptive Trigeminal Inhibition Clenching Suppression System, Pain: Pain measured at rest, Fct: Functional limitations, MO: Maximal Opening range of motion, QoL: Quality of Life.  *: Included in meta-analyses  ⁑:Interquartile range (75^th^-25^th^ percentile) | USA | University clinic | 63  61 | 88.5 %  81.0 % | 37.4 (4.2) 38.0 (3.6) | Mixed | Dental hygienists, Dentists | G1: ED and SM  G2: ED and SM + Multimodal | Biopsychosocial model, chronic pain, etiology, management methods and rationale, breathing, relaxation, emotions, stress management, self-monitoring, relapse prevention; Guided reading; 3x 50-75 min sessions + 2 phone follow-ups | Pain | 6 months  1 year | At 6 months and 1 year, G1 and G2 achieved a significant reduction in pain (p<0.05);  G1 showed a significantly greater reduction in pain at 1 year (p=0.036). |
| Fetai et al., 2021 ^a^ | Croatia | Private clinic | 21  21 | 62.0 %  62.0 % | 16-67 † | Myofascial pain  Chronic | NS | G1: ED and SM  G2: LT | Self-massage; NS; 1 session | Pain Fct | 4 weeks  3 months | At 4 weeks and 3 months, both groups demonstrated a significant reduction of pain and Fct;  G2 showed a significantly greater reduction in pain (p<0.001) and Fct (p<0.049) than G1. |
| Gebska et al., 2023 | Poland | University clinic | 28 27  27  26  26  26  26 | 100 % | 20-45 † | Myofascial pain with limited mouth opening | Physiotherapists | G1: ED and SM  G2: ED and SM + massage  G3: ED and SM + post-isometric relaxation  G4: ED and SM + Pressure point release  G5: Magneto-stimulation  G6: Magneto-ledotherapy  G7:Magneto-laserotherapy | Exercises (mobility, coordination); Written instructions; 1 session | Pain | 10 days | At 10 days, only G2, G3 and G4 achieved significant reductions in pain (p<0.001); |
| Gebska et al., 2024 | Poland | University clinic | 32  32 | 100 % | 20-45 (25.5-39.5) † | Myofascial pain with limited mouth opening  Chronic | Dentists | G1: ED and SM  G2: ED and SM + KT | Exercises (coordination, mobility, self-massage, cervical spine mobility); Written instructions; 1 session | Pain | 6 days 12 days | At 12 days, both groups demonstrated a significant reduction of pain (p<0.05);  G2 showed a significantly greater reduction in pain (p<0.001) than G1. |
| Hasanoglu E. et al., 2017 ^c^ | Turkey | University clinic | 20  20 | 85.0 % 80.0 % | 32.3 (11.9) 24.6 (9.2) | Myofascial pain | NS | G1: ED and SM  G2: ED and SM + Splint (NTI-tss) | Guidance, assurance, counseling, behavioral changes; Oral instructions; 1session | Pain  Fct | 3 weeks  6 weeks | At 3 and 6 weeks, G1 and G2 achieved significant reductions in pain (p<0.05). At 6 weeks, G1 and G2 achieved significant improvements in Fct;  No differences between groups were found for pain and Fct at 6 weeks (p=0.922, p=0.927). |
| Justribo-Manion et al., 2025 ^b^ | Spain | Hospital clinic | 17 17 | 82.4 %  82.4 % | 49.8 (18.2) 25.1 (5.1) | Mixed  Chronic | Dentists | G1: MT + ED and SM  G2: MT | Physiopathology and natural course, pain neurosciences education (PNE), changing pain-related behaviors, exercises and objectives (tongue position and mobility, jaw mobility, coordination, stretch, bitting, cervical flexors strength); Power point presentation; 2x 30 min sessions | Pain Fct | 3 weeks 5 weeks 7 weeks **19 weeks** (5 months) | At 19 weeks, both groups demonstrated a significant reduction of pain (p<0.001);  There was no significant difference between the groups in pain (p=0.4). |
| Kalamir et al., 2013 ^a^ | Australia | Private clinic | 23  23 | 65.2 % 60.9 % | 26.8 (NS) 28.2 (NS) | Myofascial pain  Chronic | Chiropractors | G1: ED and SM  G2: MT | Anatomy, biomechanics, pathophysiology, stress, breathing exercises, relaxation awareness, diet (nuts, chewing gum, etc.), exercises (stretching, self-massage, coordination, mobility, strength); Oral and written instructions, 10 sessions | Pain | 6 weeks | At 6 weeks, G1 and G2 achieved significant reductions in pain;  G2 showed a significantly greater reduction in pain than G1 (p<0.001), however this difference was not clinically important. |
| Katyayan et al., 2014 ^c^ | India | Public college and hospital | 40  40 | 77.5 % | 34.4 (20-56)† | Mixed  Chronic | Dentists | G1: ED and SM  G2: ED and SM + Splint | Exercise program (active mobility, resistance, stretching); Oral and written instructions, 1 session | Pain | 6 months | At 6 months, G1 and G2 achieved significant reductions in pain (p<0.001);  No significant differences between groups were found for pain reductions at 6 months (p=0.182). |
| Kokkola et al. 2018 | Finland | University clinic | 13  27 | 84.6 % 81.5 % | 41.0 (13.4) 47.0 (14.8) | Mixed | Dentists | G1: ED and SM  G2: ED and SM + Splint | Counselling, exercise program (active mobility, resistance, stretching); Oral and written instructions, 1 session | HRQoL | 3 months 6 months 1 year | At all time points, G1 and G2 achieved significant improvements in HRQoL (p<0.05);  No significant differences between groups for HRQoL at any time points (p=0.403, p=0.282, p=0.699). |
| Lam et al., 2020 | Sweden | Public clinic | 20  23 | 83.0 % 75.0 % | 27 (23-38)† 27 (23-37)† | Mixed  Chronic | Dentists | G1: ED and SM  G2: Splint | Goal setting, etiology, epidemiology, TMD pain, anatomy, pain physiology, acute and chronic pain, the link between quality of life and TMD pain. Treatment alternatives (analgesics, relaxation, jaw exercises, different splints, acupuncture, massage). Stress and pain, breathing exercises, diet, sleep, training, time management, setting boundaries and acceptance, setbacks and maintenance planning; Web-based program. | Pain  Fct | 3 months  6 months | At 6 months, only G2 achieved significant reductions in pain (p<0.05). G1 and G2 achieved statistically significant reductions in Fct (p<0.05);  No significant differences between groups for pain and Fct at any time points (p≥0.05). |
| Lindfors et al., 2020 | Sweden | Public clinic | 35 33 | 86.0 %  79.0 % | 33.2 (18.7) 32.7 (17.7) | Mixed  Chronic | NS | G1: ED and SM G2: Splint | Exercise program (relaxation, active mobility, resistance, stretching); Oral and written instructions, 1 session | Pain Fct | 3 months | At 3 months, both groups demonstrated a significant reduction in pain;  No significant differences between groups were found for pain reductions and Fct improvements. |
| Magesty et al., 2021 ^c^ | Brazil | University clinic | 37  36 | 68.4 % | 22.9 (7.3) | DDWR | NS | G1: ED and SM  G2: ED and SM + Unsupervised exercises | Diet, overload the masticatory muscles (chewing gum, yawning, yelling, singing, and long sessions at the dentist), parafunctions (biting nails and pencils), thermotherapy, teeth separated, posture, sleep, caffeine intake; Oral and written instructions, 1 session | HRQoL | 4 weeks | At 4 weeks, only G2 achieved a significant improvement in HRQoL (p<0.001);  G2 showed a significantly greater improvement than G1 in HRQoL (p<0.001). |
| Melo et al., 2020 ^a,c^ | Brazil | University clinic | 19  24  21  25 | 82.1 % | 28.0 (9.3) | Mixed  Chronic | NS | G1: ED and SM  G2: Splint  G3: Multimodal  G4: ED and SM + Splint | Etiology, oriented guidelines according to personal needs, characteristics of TMD, diet, physical exercises, habits, mandibular function, posture, sleep; Oral and written instructions, 2 sessions | Pain | 4 weeks | At 4 weeks, all groups achieved significant reductions in pain (p<0.001);  No statistically significant differences between groups for pain at 4 weeks (p=0.260). |
| Michelotti et al., 2004 ^c^ | Italy | University clinic | 34  36 | 91.2 % 86.1 % | 31.8 (13) 28.2 (8.8) | Myofascial pain  Chronic | Dentists | G1: ED and SM  G2: ED and SM + Unsupervised exercises | General information about self-care, etiology, prognosis, normal jaw function, overuse of the muscles, parafunctions, excessive movement, diet, teeth apart, chronic pain and psychosocial distress; Oral instructions, 4 sessions (1^st^: 60 min, 2^nd^-4^th^ : 15 min). | Pain | 3 months | At 3 months, G1 and G2 achieved significant reductions in pain (p<0.05).  No differences between groups were found for pain (p≥0.05). |
| Michelotti et al., 2012 | Italy | University clinic | 23  21 | 82.6 % 71.4 % | 30.2 (13.0) 30.3 (11.4) | Myofascial pain  Chronic | Dentists | G1: ED and SM  G2: Splint | General information about self-care, etiology, prognosis, normal jaw function, overuse of the muscles, parafunctions, excessive movement, diet, teeth apart, chronic pain and psychosocial distress; Oral instructions, 5 sessions (15 min). | Pain | 3 months | At 3 months, only G1 achieved significant reductions in pain (p=0.017).  G1 showed a significantly greater reduction in pain at 3 months (p=0.034). |
| Mulet et al., 2007 ^c^ | USA | University clinic | 22  20 | 95.5 % 95.0 % | 23.4 (2.1) 25.1 (2.3) | Myofascial pain  Chronic | Physiotherapists | G1: ED and SM  G2: ED and SM + Unsupervised exercises | Optimistic counseling, reassurance, rest the masticatory muscles, thermotherapy, maladaptive behaviors (tooth clenching, grinding, caffeine intake, gum chewing, stomach sleeping, resting the jaw on the hand, wide opening of the mouth, diet, bilateral chewing, calcium intake); Oral and written instructions; 1 session + 1 in-person follow-up. | Pain | 1 week  4 weeks | At 4 weeks, G1 and G2 achieved a significant reductions in pain (p<0.001);  No significant differences between groups were found for pain at 4 weeks (p=0.82). |
| Nagata et al., 2019 | Japan | University clinic | 30 31 | 82.0 % | 50.7 (18.3) 48.2 (21.1) | Mixed with limited mouth opening | Dentists | G1: ED and SM  G2: ED and SM + MT (manipulation) | TMD self-management, pain and stress, diet, parafunctions, posture, clenching, exercise program (stretching, myo-functional therapy); Oral instructions; 1 session | Pain | 10 weeks 18 weeks | At 10 weeks, both groups demonstrated a significant reduction of pain;  No significant difference between the groups in pain at 10 weeks (p>0.05). |
| Niemelä et al., 2012 ^c^ | Finland | University clinic | 41  39 | 73.2 % 82.1 % | 44.1 (13.1) 43.2 (13.3) | Mixed | Dentists | G1: ED and SM  G2: ED and SM + Splint | Exercise program (relaxation, active mobility, resistance, stretching); Oral and written instructions; 1 session | Pain | 4 weeks | At 4 weeks, G1 and G2 showed significant reductions in pain;  No significant differences between groups were found for pain at 4 weeks (p≥0.05). |
| Olbort et al., 2023 | Germany | Dental practice | 30 30 | 60%  80% | 48.0 (17.9) 50.7 (14.8) | DDWR | Doctor | G1: ED and SM  G2: Splint | Exercise program (coordination, stretching, strength); Oral and video instructions; 1 session | Pain | 2 months 4 months **6 months** | At 6 months, both groups demonstrated a significant reduction of pain (p<0.001);  At 6 months, there was no difference between the groups in pain (p = 0.092). |
| Patil et al,. 2017 | Kingdom of Saudi Arabia | Dentistery college | 18 18 | 66.6 %  61.1 % | 34.0 (7.0) 32.9 (12.6) | Mixed | NS | G1: ED and SM  G2: TENS | Management of TMD, lifestyle, ergonomic changes, exercise program (active and passive mobility, resistive, stretching, strength); Oral instructions; 1 session | Pain on palpation | 1 week 2 weeks 3 weeks **4weeks** | At 4 weeks, both groups demonstrated a significant reduction of muscle pain;  G2 showed a significantly greater reduction of muscle pain than G1 at 4 weeks (p<0.05). |
| Pehlivan et al., 2024 ^b^ | Turkey | Rehabilitation hospital | 10 10 | 70 %  90 % | 31.4 (11.2) 35.3 (18.1) | Mixed  Chronic | Physiotherapists | G1: Multimodal + ED and SM  G2: Multimodal | Parafunctions, pain management, exercise program (tongue position, posture, head posture, active mobility, coordination, stabilization); Oral and written instructions; 1 session | Pain HRQoL | 3 weeks | At 3 weeks, both groups demonstrated a significant reduction of pain (p<0.05). HRQoL improved in G1 for all domains but improved only for bodily pain domain in G2;  G1 showed a significantly greater reduction in pain and HRQoL than G1 (p<0.05). |
| Peixoto et al., 2021 ^a^ | Brazil | University clinic | 24  20  19  18 | NS | 18-65 | Mixed | Dentists | G1: ED and SM  G2: Splint  G3: Multimodal  G4: Acupuncture | General characteristics, self-management, diet, physical exercises, harmful habits, posture, sleep hygiene; Oral and written instructions; 1 session | Pain  HRQoL | 4 weeks | At 4 weeks, all groups achieved significant reductions in pain (p=0.015).  G3 showed a significantly greater reduction in pain than G1 at 4 weeks (p=0.004). No other differences between groups were found for pain and HRQoL (p≥0.05). |
| Qvintus et al., 2015 ^c^ | Finland | University clinic | 41  37 | 73.2 % 83.8 % | 44.0 (13.1) 42.6 (13.4) | Mixed | Dentists | G1: ED and SM  G2: ED and SM + Splint | Exercise program (active mobility, resistance, stretching); Oral and written instructions, 1 session | Pain | 1 year | At 1 year, G1 and G2 showed a reduction in pain; No differences between groups were found for pain at 1 year. |
| Ram et al., 2021 ^c^ | India | NS | 40  40  40  40 | 55 % 42.5 % 65 % 55% | 37.5 (10.2) 37.6 (10.6) 42.3 (9.9) 40.4 (10.4) | Mixed | Dentists | G1: ED and SM  G2: ED and SM + Muscle energy therapy  G3: ED and SM + Splint  G4: ED and SM + Muscle energy therapy + Splint | Diagnosis, prognosis, reassurance, biopsychosocial etiology, sleep, use of analgesics, anatomy, jaw function, parafunctions, (clenching, clicking, grinding), unilateral gum chewing, sleep, breathing exercises, diet, thermotherapy; Oral instructions; 1 session | Pain | 1 week  2 weeks  4 weeks  3 months | At 2 weeks, 1 and 3 months, all groups achieved a significant reduction in pain (p<0.05);  G2, G3 and G4 showed a greater reduction in pain than G1 at 1 and 3 months (p<0.001). |
| Salloum et al., 2024 ^a^ | Syria | University clinic | 20 20 20 20 | 80 %  20 %  70 %  70% | 29.9 (7.5) 31.2 (8.3) 31.0 (9.0) 28.2 (6.2) | Myofascial pain with limited mouth opening | NS | G1: ED and SM  G2: TheraBite Device  G3: Multimodal physiotherapy (thermotherapy, US, MT)  G4: Splint | Exercise program (active mobility, resistive movement, coordination); Oral and written instructions; 1 session | Pain | 4 weeks 5 months | All groups demonstrated a significant reduction of pain at all time-points;  At 4 weeks, G2, G3 and G4 showed a greater reduction of pain than G1 (p=0.046, p=0.001, p=0.002) |
| Seyhan et al., 2023 ^c^ | Turkey | University clinic | 20 20 | 90 % 90 % | 26.1 (8.5) 41.1 (15.0) | Myofascial pain | Physiotherapists | G1: ED and SM  G2: ED and SM + Supervised exercises | Exercise program (tongue position, posture, head posture, active mobility, coordination, stabilization); Oral instructions; 1 session | Pain Fct HRQoL | 6 weeks | Both groups demonstrated a significant reduction of pain (p=0.000; p=0.002) and Fct (p=0.010; p=0.012) and improvement of HRQoL (p=0.001);  No significant difference between the groups in pain (p = 0.745), Fct (p=0.685) and HRQoL (p=0.871) after treatment. |
| Shah et al., 2024 | Pakistan | Hospital | 20 20 | 55 %  65 % | 37.3 (8.3) 37.9 (7.4) | Mixed | Physiotherapists | G1: ED and SM  G2: ED and SM + MT | Etiology, pain, diet, caffeine intake, hydratation, posture, muscle hyperactivity, breathing exercises, relaxation, exercise program; Oral instructions; 1 session | Pain | 4 weeks | At 4 weeks, both groups demonstrated a significant reduction in pain;  G2 showed a significantly greater reduction of pain than G1 at 4 weeks (p<0.001). |
| Simões et al., 2023 | Brazil | University clinic | 36  36 | 68.4% | 25.9 (7.3) | DDWR | NS | G1: ED and SM  G2: ED and SM + Unsupervised exercises | Diet, overload muscles (chewing gum, yawning, yelling, singing, long sessions at dentist), thermotherapy, teeth separated, posture, sleep, parafunctions, caffeine intake; Oral and written instructions; 1 session | Pain on palpation | 24 hours  1 week  4 weeks | At 4 weeks, G1 and G2 achieved a significant reduction in pain;  G2 showed greater reductions in pain than G1 at 24 hours and 4 weeks (p<0.05). |
| Tanhan et al., 2023 ^c^ | Turkey | NS | 17  20  22 | 76.5 %  65 %  90.9 % | 20.5 (1.5) 21.4 (4.3) 21.0 (1.8) | Myofascial pain | Physiotherapists | G1: ED and SM  G2: ED and SM + LT  G3: ED and SM + MT | Parafunctions, psychological factors, pain, self-management, exercise program (tongue position, resistive movement, coordination, chin tucks, body posture, cervical stretching and strengthening); Oral and written instructions; 1 session | Pain in activity | 4 weeks | At 4 weeks, all groups achieved a significant reduction in pain (p<0.01);  No significant differences between groups were found in pain at 4 weeks (p>0.05). |
| Tavera et al., 2012 | Mexico | Institute for Clinical Research | 28 60 64 | 89.3 %  80.0 %  82.8 % | 36.3 (13.0) 37.3 (10.6) 38.0 (11.0) | Mixed | Dentists | G1: ED and SM  G2: TMDes  G3: Splint | Exercise program (active mobility, stretching), thermotherapy; Oral instructions; 1 session | Pain | 4 weeks  2 months  3 months | All groups demonstrated a significant reduction of pain at all time-points;  No significant difference between the groups in pain at all time-points. |
| Truelove et al., 2006 | USA | University clinic | 64  68  68 | 81 % 87 %  90 % | 36 (11) 36 (11) 35 (12) | Mixed | Dentists | G1: ED and SM  G2: ED and SM + Splint (Hard acrylic)  G3: ED and SM + Splint (Soft vinyl) | Jaw relaxation, parafunctions, thermotherapy, NSAIDs, stress reduction, self-care strategies, exercise (opening stretches); Oral instructions; 1 session | Pain  Fct | 3 months  6 months  1 year | At 1 year, G1-G3 achieved a significant reduction in pain, and improvements in Fct (p<0.0001);  No significant differences between groups were found for pain and Fct at 1 year. |
| Tuncer et al., 2013 ^c^ | Turkey | University clinic | 20  20 | 75 % 80 % | 34.8 (12.4) 37.0 (14.6) | Mixed | Physiotherapists | G1: ED and SM  G2: ED and SM + MT | Etiology, ergonomic advice, breathing, relaxation, posture, exercise program (active mobility, stretching, resistive movement); Oral instructions; 1 session | Pain | 4 weeks | At 4 weeks, G1 and G2 achieved a significant reductions in pain (p<0.001);  No significant differences between groups we found for pain at 4 weeks. |
| Ucar et al., 2014 | Turkey | University clinic | 18  20 | 61.1 % 60.0 % | 29 (10) 27 (11) | Myofascial pain | Physiotherapists | G1: ED and SM  G2: ED and SM + US | Lifestyle change, coping mechanisms, ergonomic regulation, exercise program (active mobility, stretching, resistive movement); Oral instructions; 1 session | Pain | 2 weeks | At 2 weeks, G1 and G2 achieved significant reductions in pain (p<0.05);  G2 showed a significantly greater reduction in pain (p<0.05) than G1 at 2 weeks. |
| Wanman et al., 2018 ^a^ | Sweden | University clinic | 30 30 30 | 70.0 % | 39.2 (15.2) | DDWR | NS | G1: ED and SM  G2: Supervised exercises  G3: Splint | Anatomy and function, diet, avoid clicking, exercise program (active mobility, resistive movement); Oral and written instructions; 1 session + 1 in-person follow-up. | Pain Fct | 3 months | At 3 months, all groups demonstrated a significant reduction of pain (p<0.001) and Fct (p<0.001);  No significant difference between the groups in pain and Fct at 3 months. |
| Wright et al., 2000 ^c^  RDC/TMD: Research Diagnostic Criteria for Temporomandibular Disorders, NS: Not specified, LLLT: Low-Level-Laser Therapy MT: Manual Therapy, US: Ultrasound therapy, Pain: Pain measured at rest, QoL: Quality of Life,  MO: Maximal Opening range of motion, Fct: Functional Limitations.  *: Included in meta-analyses | USA | Air Force Base clinic | 30  30 | 83.3 %  86.7 % | 30.8 32.7 | Myofascial pain Chronic | Physiotherapists | G1: ED and SM  G2: ED and SM + Supervised exercises | Rest masticatory muscles, parafunctions, thermotherapy, NSAIDs; Oral instructions; 1 session | Pain | 4 weeks | At 4 weeks, only G2 achieved a significant reduction in pain (p<0.05);  G2 showed a significantly greater reduction in pain (p<0.001) than G1 at 4 weeks. |

† Expressed in Min – Max (Interquantile range)

Diagnostic categories were based on the RDC/TMD or DC/TMD: Diagnostic Criteria for Temporomandibular Disorders (most recent version of RDC/TMD)(ref)

**Abbreviations.** n: Number of participants; SD: Standard deviation; TMD: Temporomandibular disorders; ED and SM: Education and self-management interventions; NS: Not specified, Fct: functional limitations; HVES: High voltage electric stimulation; NTI-tss: Nociceptive Trigeminal Inhibition Clenching Suppression System; DDwR: Dis displacement without reduction; HRQoL: Health-related quality of life; LT: Laser therapy; KT: Kinesiotaping; MT: Manual Therapy, DDWR: Disc displacement with reduction, TENS: ; TMDes: Temporomandibular ear system; US: Ultrasound therapy.

^a^ included in meta-analyses that compared ED and SM to other interventions

^b^ included in meta-analyses that compared other interventions combined with ED and SM compared to that other intervention alone

^c^ included in meta-analyses that compared ED and SM combined with any other interventions compared to ED and SM alone.
