# Supplementary material for: Patient Education and Self‐Management in Adults With Temporomandibular Disorders: Results From a Systematic Review With Meta‐Analysis
Source: J Oral Rehabil. 2026 Mar 19;53(7):1394–408. doi: 10.1111/joor.70187 (PMC13261784; doi:10.1111/joor.70187)
Supplement: Supplementary file 4 — File S4: Details on the ED and SM interventions. [file JOOR-53-1394-s004.docx]

|  |  | aksu et al. 2019 | arikan et al. 2025 | benli et al. 2024 | benlidayi et al., 2016 | brandão et al., 2002 | carlson et al., 2001 | conti et al., 2012 | conti et al., 2015 | craane et al. 2011 | craane et al., 2012 | denordenflytch et al. 2024 | de resende et al., 2021 | dworkin et al., 2002 | fetai et al. 2021 | gebska et al., 2023 | gebska et al. 2024 | hasanoglue. et al., 2017 | justribo-manion et al. 2024 | kalamir et al., 2013 | katyayan et al., 2014 | kokkola et al., 2018 | lam et al., 2020 | lindfors et al. 2020 | magesty et al., 2021 | melo et al., 2020 | michelotti et al., 2004 | michelotti et al.,2012 | mulet et al., 2007 | nagata et al. 2018 | niemelä et al., 2012 | olbort et al. 2023 | patil et al. 2017 | pelhivan et al. 2024 | peixoto et al., 2021 | qvintus et al.,2015 | ram et al., 2021 | salloum et al. 2024 | seyhan et al. 2023 | shah et al., 2024 | simões et al., 2023 | tanhan et al., 2023 | tavera et al. 2012 | truelove et al., 2006 | tuncer et al., 2013 | ucar et al., 2014 | wanman et al. 2018 | wright et al., 2000 | number of studies |
| --- | --- | --- | --- | --- | --- | --- | --- | --- | --- | --- | --- | --- | --- | --- | --- | --- | --- | --- | --- | --- | --- | --- | --- | --- | --- | --- | --- | --- | --- | --- | --- | --- | --- | --- | --- | --- | --- | --- | --- | --- | --- | --- | --- | --- | --- | --- | --- | --- | --- |
| Characteristics of the education and self-management programs | self-management with no or incomplete details |  |  |  | x |  |  |  |  |  |  |  |  |  | x |  |  | x |  |  | x | x |  |  |  |  |  |  |  |  | x |  |  |  |  | x |  |  |  | x |  | x |  |  |  | x |  |  | 10 |
|  | individualized program |  |  |  |  |  |  |  |  |  |  |  | x | x |  |  |  |  |  |  |  |  |  |  |  | x |  |  |  |  |  |  |  |  |  |  |  |  | x |  |  | x |  |  |  |  |  |  | 5 |
|  | references available |  | x | x | x | x | x |  |  |  |  |  |  |  |  | x | x |  |  |  |  |  |  |  | x |  | x | x | x | x | x |  | x | x |  | x | x |  | x | x | x | x |  |  | x |  | x | x | 24 |
|  | oral instructions only |  | x |  |  | x |  |  | x |  |  |  | x |  |  |  |  | x |  |  |  |  |  |  |  |  | x |  |  | x |  |  | x |  |  |  | x |  | x | x |  |  | x | x | x | x |  | x | 16 |
|  | written or videotaped instructions | x |  |  | x |  | x | x |  | x | x | x |  | x |  | x |  |  | x | x | x | x | x |  | x | x |  | x | x |  | x | x |  | x | x | x |  | x |  |  | x | x |  |  |  |  | x |  | 27 |
| Providing general information^a^ | anatomy and physiology of tmj |  |  |  |  |  |  |  |  | x | x |  |  |  |  |  |  |  |  | x |  |  | x |  |  | x | x | x |  |  |  |  |  |  | x |  | x |  |  |  |  |  |  |  |  |  |  |  | 9 |
|  | etiology of tmd (behavioral, emotional) |  |  |  |  |  | x |  |  | x | x | x | x | x |  |  | x |  |  |  |  |  | x |  |  | x | x | x |  |  |  |  |  |  |  |  | x |  | x | x |  |  |  |  | x |  |  |  | 15 |
|  | reassurance about tmd symptoms |  |  |  |  |  |  |  |  |  |  |  |  |  |  |  |  |  | x |  |  |  |  |  |  |  |  |  | x |  |  |  |  |  |  |  | x |  |  |  |  |  |  |  |  |  |  |  | 3 |
|  | explanation of diagnostic and prognosis |  |  |  |  |  |  |  |  |  |  | x |  |  |  |  |  |  | x |  |  |  |  |  |  | x | x | x |  |  |  |  |  |  |  |  | x |  | x |  |  |  |  |  |  |  |  |  | 7 |
|  | pain physiology |  |  |  |  |  | x |  |  |  |  |  |  | x |  |  |  |  | x |  |  |  | x |  |  |  |  |  |  |  |  |  |  | x |  |  |  |  |  |  |  |  |  |  |  |  |  |  | 5 |
| Avoiding overuse of the masticatory system^a^ | keeping the jaw muscles relaxed |  | x |  |  |  | x | x | x | x | x |  | x |  |  |  | x |  |  |  |  |  |  |  |  |  | x |  | x | x |  |  |  |  |  |  | x |  |  | x | x |  |  | x |  |  |  | x | 16 |
|  | diet modification | x | x |  |  | x | x | x | x |  |  | x |  |  |  |  |  |  |  | x |  |  | x |  | x | x | x | x | x | x |  |  |  |  | x |  | x |  |  | x | x |  |  |  |  |  |  |  | 19 |
|  | avoid non-tmj specific parafunctional habits (i.e neck, head or shoulder) |  | x |  |  |  | x |  |  |  |  |  |  |  |  |  |  |  |  |  |  |  |  |  |  |  |  |  |  |  |  |  |  |  |  |  |  |  |  |  |  |  |  |  |  |  |  |  | 2 |
|  | avoid oral parafunctions (bitting pens, nails, mouth corners, chewing gum) |  | x |  |  | x |  |  |  | x |  | x | x |  |  |  | x |  |  |  |  |  |  |  | x | x | x | x | x | x |  |  |  | x | x |  | x |  | x |  | x | x |  | x |  |  |  | x | 20 |
|  | avoid excessive opening mouth |  | x |  |  | x |  |  |  | x | x |  | x |  |  |  |  |  |  |  |  |  |  |  | x |  | x | x | x |  |  |  |  |  |  |  |  |  | x |  | x |  |  |  |  |  |  |  | 11 |
|  | bilateral chewing |  |  |  |  |  |  |  |  |  |  |  |  |  |  |  |  |  |  |  |  |  |  |  |  |  |  |  | x |  |  |  |  |  |  |  | x |  |  |  |  |  |  |  |  |  |  |  | 2 |
| Posture education^a^ | tongue and mandibular rest position |  | x |  |  |  |  |  |  |  |  |  |  |  |  |  |  |  | x |  |  |  |  |  | x | x | x |  | x |  |  |  |  |  |  |  |  |  | x | x | x | x |  |  |  |  |  |  | 10 |
|  | maintain a good body posture |  | x |  |  |  |  |  |  |  |  |  | x |  |  |  |  |  |  |  |  |  |  |  | x | x |  |  | x | x |  |  |  |  | x |  |  |  |  | x | x | x |  |  | x |  |  | x | 12 |
| Lifestyle and psychosocial factors^a^ | improving sleep hygiene |  | x |  |  |  | x | x | x |  |  | x | x |  |  |  |  |  |  |  |  |  | x |  | x | x |  |  |  |  |  |  |  |  | x |  | x |  |  |  | x |  |  |  |  | x |  |  | 13 |
|  | physical activity |  |  |  |  |  | x |  |  |  |  | x | x |  |  |  |  |  |  |  |  |  | x |  |  | x |  |  |  |  |  |  |  |  | x |  |  |  |  |  |  |  |  |  |  | x |  |  | 7 |
|  | avoid caffeinated drinks |  |  |  |  |  |  | x | x |  |  |  | x |  |  |  |  |  |  |  |  |  |  |  | x |  |  |  |  |  |  |  |  |  |  |  |  |  |  | x | x |  |  |  |  | x |  |  | 7 |
|  | proper hydratation |  |  |  |  |  |  |  |  |  |  |  |  |  |  |  |  |  |  |  |  |  |  |  |  |  |  |  |  |  |  |  |  |  |  |  |  |  |  | x |  |  |  |  |  |  |  |  | 1 |
|  | help identify psychosocial factors and how they may contribute to complaints |  | x |  |  |  |  |  |  |  |  |  |  | x |  |  |  |  |  | x |  |  | x |  |  |  | x | x |  |  |  |  |  |  |  |  |  |  |  |  |  | x |  |  |  | x |  |  | 8 |
| Exercise and thermotherapy^a^ | self-massage | x |  |  |  |  |  | x |  |  |  | x |  |  |  |  | x |  |  | x |  |  |  |  |  |  | x |  |  |  |  |  |  |  |  |  |  |  |  |  |  |  |  |  |  |  |  |  | 6 |
|  | heat application | x |  |  |  |  |  | x |  |  |  |  |  |  |  |  |  |  |  |  |  |  |  |  |  |  | x |  | x |  |  |  |  |  |  |  | x |  |  |  | x |  | x | x |  |  |  | x | 9 |
|  | ice application | x |  |  |  |  |  |  |  |  |  |  |  |  |  |  |  |  |  |  |  |  |  |  |  |  |  |  | x |  |  |  |  |  |  |  |  |  |  |  | x |  |  |  |  |  |  |  | 3 |
|  | self-monitoring and awareness |  | x |  |  |  |  |  |  |  |  |  |  |  |  |  |  |  |  |  |  |  |  |  |  |  |  | x |  |  |  |  |  |  |  |  | x |  |  |  |  |  |  |  |  |  |  | x | 4 |
|  | cervical exercises |  |  |  |  |  |  |  |  |  |  |  |  |  |  |  | x |  | x |  |  |  |  |  |  |  |  |  | x |  |  |  |  |  |  |  |  |  | x |  |  | x |  |  |  |  |  | x | 6 |
|  | upper back exercises |  |  |  |  |  |  |  |  |  |  |  |  |  |  |  |  |  |  |  |  |  |  |  |  |  |  |  |  |  |  |  |  |  |  |  |  |  | x |  |  |  |  |  |  |  |  | x | 1 |
|  | stretching |  | x | x |  |  |  |  |  |  |  | x |  |  |  |  |  |  |  | x | x | x |  | x |  |  | x |  |  | x | x |  | x |  |  | x |  |  |  | x |  |  | x | x | x | x |  |  | 17 |
|  | strengthening |  | x |  | x |  |  |  |  |  |  | x |  |  |  |  |  |  |  | x | x | x |  | x | x |  |  |  | x |  | x | x | x |  |  | x |  | x | x | x |  | x |  |  | x | x |  |  | 19 |
|  | active rom |  | x | x | x |  |  |  |  |  |  |  |  |  |  | x | x |  | x | x | x | x |  | x | x |  |  |  |  | x | x |  | x |  |  | x |  | x |  | x |  |  |  |  | x | x |  |  | 19 |
|  | coordination |  | x | x |  |  |  |  |  |  |  |  |  |  |  | x | x |  | x |  |  |  |  |  | x |  | x |  | x |  |  | x |  |  |  |  |  | x | x | x |  | x |  |  |  |  |  |  | 13 |
|  | relaxation and breathing exercises | x | x |  |  |  | x | x | x |  |  |  |  | x |  |  |  |  |  | x |  |  | x |  |  |  | x |  | x |  |  |  |  |  |  |  | x |  |  | x |  |  |  |  | x |  |  |  | 13 |
| Additional self-management information^a^ | use of analgesics |  |  |  |  |  |  |  |  |  |  |  |  |  |  |  |  |  |  |  |  |  | x |  |  |  |  |  |  |  |  |  |  |  |  |  | x |  |  |  |  |  |  | x |  |  |  | x | 4 |
|  | communication with health providers |  |  |  |  |  |  |  |  |  |  |  |  | x |  |  |  |  |  |  |  |  |  |  |  |  |  |  |  |  |  |  |  |  |  |  |  |  |  |  |  |  |  |  |  |  |  |  | 1 |
|  | maintenance and relapse prevention |  |  |  |  |  |  |  |  |  |  |  |  | x |  |  |  |  |  |  |  |  | x |  |  |  |  |  |  |  |  |  |  |  |  |  |  |  |  |  |  |  |  |  |  |  |  |  | 2 |

^a^Components of education and self-management as described by van der Meer et al. (2024)

|  |
| --- |
