# Supplementary material for: Patient Education and Self‐Management in Adults With Temporomandibular Disorders: Results From a Systematic Review With Meta‐Analysis
Source: J Oral Rehabil. 2026 Mar 19;53(7):1394–408. doi: 10.1111/joor.70187 (PMC13261784; doi:10.1111/joor.70187)
Supplement: Supplementary file 5 — File S5: Table of the descriptive parameters of ED and SM. [file JOOR-53-1394-s002.docx]

| **Education and self-management** | | | | | **Other non-surgical interventions** | | | | |
| --- | --- | --- | --- | --- | --- | --- | --- | --- | --- |
|  | Min | Max | Median (IQR) | Mean (SD) |  | Min | Max | Median (IQR) | Mean (SD) |
| Duration of each session (minutes) (n=6) | 15 | 75 | 40.0 (27.5) | 43.3 (22.3) | Duration of each session (minutes) (n=24) | 5 | 75 | 30 (27.8) | 34.8 (18.4) |
| Number of in-person sessions  (n=46) | 1 | 15 | 1.0  (1.75) | 2.3 (2.7) | Number of in-person sessions  (n=47) | 1 | 20 | 4 (9) | 6.1 (5.3) |
| Number follow-ups (n=46) | 0 | 4 | 0.0 (0.0) | 0.4 (1.0) | Number follow-ups (n=47) | 0 | 7 | 0.0 (0.0) | 0.5 (1.3) |
| Total duration  (duration x frequency)  (minutes)  (n=6) | 60 | 195 | 87.5 (51.3) | 101.7 (51.4) | Total duration  (duration x frequency)  (minutes)  (n=24) | 30 | 825 | 191 (230) | 247.1 (207.3) |

**Abbreviations.** Min: Minimum range; Max: Maximum range; IQR : Inter-quantile difference (75th-25th); SD: Standard deviation
