# Supplementary material for: Patient Education and Self‐Management in Adults With Temporomandibular Disorders: Results From a Systematic Review With Meta‐Analysis
Source: J Oral Rehabil. 2026 Mar 19;53(7):1394–408. doi: 10.1111/joor.70187 (PMC13261784; doi:10.1111/joor.70187)
Supplement: Supplementary file 6 — File S6: Cochrane Risk of Bias v1. [file JOOR-53-1394-s006.docx]

| Authors | Random Sequence Generation | Allocation concealment | Blinding (participants and personnel) | Blinding (outcome assessment) | Incomplete outcome data | Selective reporting | Other sources of bias |
| --- | --- | --- | --- | --- | --- | --- | --- |
| Aksu et al., 2019 | High ^a^ | Unclear | High ^c^ | High ^d^ | Low | Low | Low |
| Arikan et al., 2024 | High ^a^ | Unclear | High ^c^ | Unclear | Unclear | Low | Low |
| Benli et al., 2024 | Low | Unclear | High ^c^ | Low | High ^e^ | High ^f^ | Unclear |
| Benlidayi et al., 2016 | Unclear | Low | High ^c^ | Low | Low | Low | Low |
| Brandão et al., 2002 | Low | Unclear | High ^c^ | High ^d^ | Low | Low | Low |
| Carlson et al., 2001 | Low | Unclear | High ^c^ | Low | High ^e^ | Low | Low |
| Conti et al., 2012 | Unclear | Unclear | High ^c^ | Low | High ^e^ | Unclear | Low |
| Conti et al., 2015 | Unclear | Unclear | High ^c^ | Low | High ^e^ | Unclear | Low |
| Craane et al., 2011 | Low | Low | High ^c^ | Low | Low | Low | Low |
| Craane et al., 2012 | Low | Low | High ^c^ | Low | Low | Low | Low |
| DeNordenflycht et al., 2024 | Low | Unclear | Unclear | Low | Low | Low | Low |
| de Resende et al., 2021 | Low | Unclear | High ^c^ | Low | Low | High ^f^ | Low |
| Dworkin et al., 2002 | Unclear | Unclear | High ^c^ | Unclear | High ^e^ | Low | Low |
| Fetai et al., 2021 | Low | Unclear | High ^c^ | Unclear | High ^e^ | Low | Unclear |
| Gebska et al., 2023 | Unclear | Low | High ^c^ | Low | Low | Low | Unclear |
| Gebska et al., 2024 | Unclear | Low | High ^c^ | Low | Unclear | Low | Low |
| Hasanoglu E. et al., 2017 | Unclear | Unclear | High ^c^ | Low | Low | Low | Low |
| Justribo-Manion et al., 2024 | Low | Low | Low | Low | Low | High ^f^ | Low |
| Kalamir et al., 2013 | Low | Low | High ^c^ | Low | Low | Low | Low |
| Katyayan et al., 2014 | Low | Low | High ^c^ | Low | Low | Unclear | Unclear |
| Kokkola et al., 2018 | Low | Unclear | High ^c^ | Low | High ^e^ | Unclear | High ^g^ |
| Lam et al., 2020 | Low | Low | High ^c^ | High ^d^ | High ^e^ | Low | Low |
| Lindfors et al., 2020 | Low | Low | Low | Low | Low | Low | High ^h^ |
| Magesty et al., 2021 | Unclear | Low | High ^c^ | Low | Low | High ^f^ | Low |
| Melo et al., 2020 | Low | Unclear | High ^c^ | Low | Low | Low | Low |
| Michelotti et al., 2004 | Low | Unclear | High ^c^ | Low | High ^e^ | Unclear | Low |
| Michelotti et al.,2012 | Low | Unclear | High ^c^ | Low | Low | Unclear | Low |
| Mulet et al., 2007 | Low | Unclear | Unclear | Low | Unclear | Low | Low |
| Nagata et al., 2018 | Low | Unclear | High ^c^ | Unclear | High ^e^ | Low | Unclear |
| Niemelä et al., 2012 | Low | Unclear | High ^c^ | Low | Unclear | Unclear | Low |
| Olbort et al., 2023 | Low | Unclear | High ^c^ | High ^d^ | Low | Low | Unclear |
| Patil et al., 2017 | Unclear | Unclear | High ^c^ | Unclear | Unclear | Low | Unclear |
| Pehlivan et al., 2024 | Unclear | Low | Unclear | Unclear | Unclear | Low | High ^h^ |
| Peixoto et al., 2021 | Unclear | Unclear | High ^c^ | Unclear | High ^e^ | High ^f^ | Low |
| Qvintus et al.,2015 | Low | Unclear | High ^c^ | Low | High ^e^ | Unclear | High ^g^ |
| Ram et al., 2021 | Low | Low | High ^c^ | High ^d^ | High ^e^ | Unclear | Low |
| Salloum et al., 2024 | Low | Unclear | High ^c^ | Unclear | Low | Low | Low |
| Seyhan et al., 2023 | Unclear | Low | High ^c^ | Unclear | Low | Low | Low |
| Shah et al., 2024 | Unclear | Unclear | Unclear | Low | Unclear | Low | Unclear |
| Simões et al., 2023 | Unclear | Low | High ^c^ | Unclear | Low | Low | Low |
| Tanhan et al., 2023 | Low | Unclear | High ^c^ | Unclear | High ^e^ | Low | High ^i^ |
| Tavera et al., 2012 | Unclear | Unclear | High ^c^ | High ^d^ | High ^e^ | Low | Unclear |
| Truelove et al., 2006 | Low | Low | High ^c^ | Low | Low | Unclear | Low |
| Tuncer et al., 2013 | Low | Unclear | High ^c^ | Low | Low | Unclear | Low |
| Ucar et al., 2014 | Unclear | Unclear | High ^c^ | Unclear | High ^e^ | Unclear | Low |
| Wanman et al., 2018 | Low | High ^b^ | High ^c^ | Low | Low | Low | Low |
| Wright et al., 2000 | Unclear | Unclear | High ^c^ | Low | Low | Unclear | Low |

*Green color indicates a low risk of bias, yellow indicates some concerns because of unclear information, red color indicates a high risk of bias*

^a^ Selection bias (biased allocation to interventions) due to inadequate generation of a randomised sequence.

^b^ Selection bias (biased allocation to interventions) due to inadequate concealment of allocations before assignment.

^c^ Reporting bias due to selective outcome reporting.

^d^ Risk of bias related to the fact that some participants moved from one group to another.

^e^ Risk of bias related to inappropriate sample size for statistical power.

^f^ Risk of bias related to the exclusion of non-observant patients.

^g^ Performance bias due to knowledge of the allocated interventions by participants and personnel during the study.

^h^ Detection bias due to knowledge of the allocated interventions by outcome assessment.

^i^ Attrition bias due to amount, nature, or handling of incomplete outcome data.
