# Supplementary material for: Patient Education and Self‐Management in Adults With Temporomandibular Disorders: Results From a Systematic Review With Meta‐Analysis
Source: J Oral Rehabil. 2026 Mar 19;53(7):1394–408. doi: 10.1111/joor.70187 (PMC13261784; doi:10.1111/joor.70187)
Supplement: Supplementary file 7 — File S7: Sensitivity analyses. [file JOOR-53-1394-s005.docx]

| **ED and SM compared to other interventions** |
| --- |
| **Comparator: Any other interventions on short-term pain (1-6 weeks)** |
| **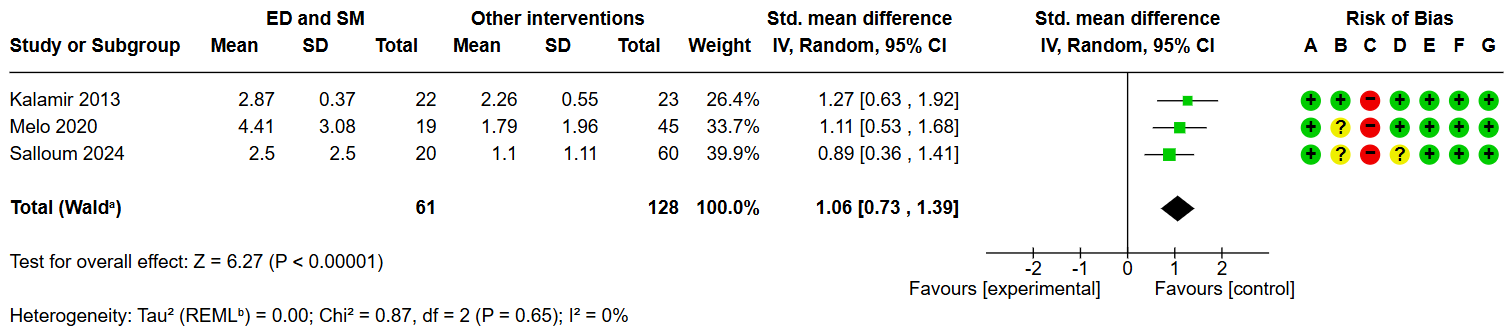** |
| **ED and DM combined with other interventions compared to ED and SM alone** |
| **With any other interventions on pain short-term (1-6 weeks)** |
| **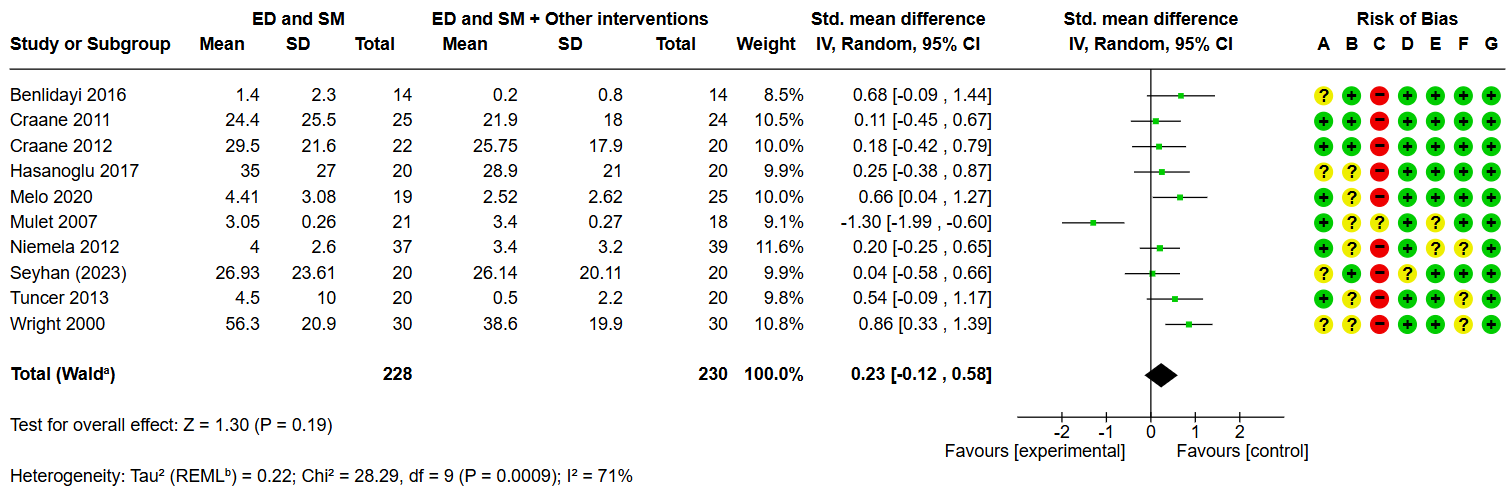** |
| **With any other interventions on medium-term pain (7-26 weeks)** |
| **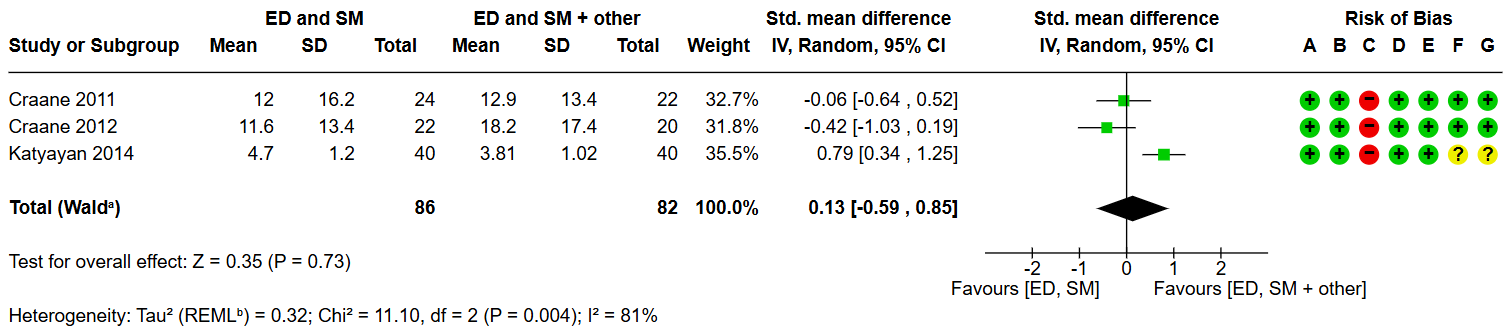** |
| **With any other interventions on short-term function (1-6 weeks)** |
| 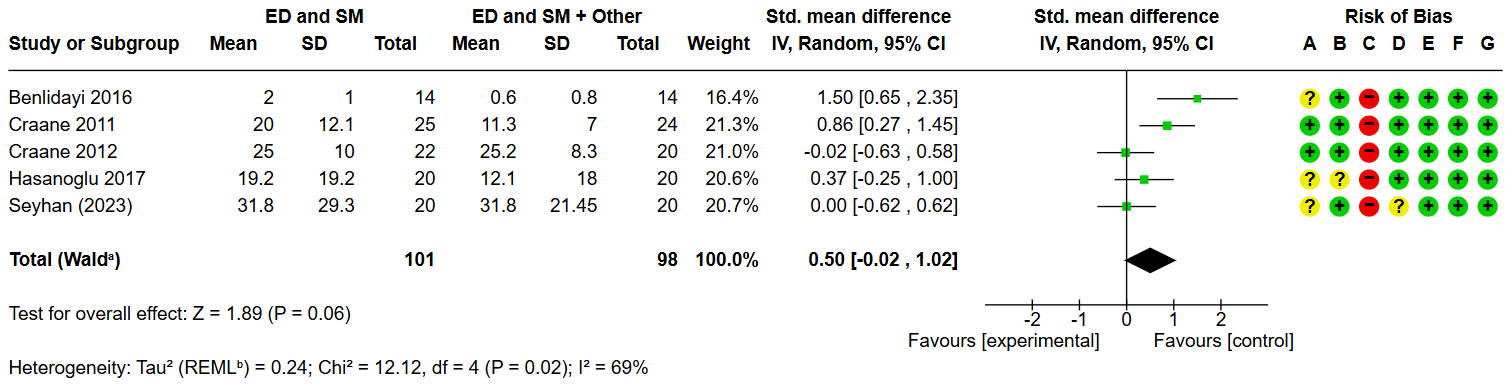 |
